# 1,2,4-Triphospholyl anions – versatile building blocks for the formation of 1D, 2D and 3D assemblies†
†Dedicated to Professor Ekkehardt Hahn on the occasion of his 60th birthday.
‡
‡Electronic supplementary information (ESI) available: Experimental part, crystallographic data and additional figures. CCDC 1056476–1056484. For the ESI and crystallographic data in CIF or other electronic format see DOI: 10.1039/c5dt01230a



**DOI:** 10.1039/c5dt01230a

**Published:** 2015-05-11

**Authors:** Claudia Heindl, Eugenia V. Peresypkina, Alexander V. Virovets, Vladislav Yu. Komarov, Manfred Scheer

**Affiliations:** a Institut für Anorganische Chemie , Universität Regensburg , Universitätsstr. 31 , 93053 Regensburg , Germany . Email: Manfred.Scheer@ur.de; b A. V. Nikolaev Institute of Inorganic Chemistry , SB RAS , Ak. Lavrentiev prosp. 3 , Novosibirsk 630090 , Russia; c Novosibirsk State University , Pirogova 2 , Novosibirsk 630090 , Russia

## Abstract

The potential of K[P_3_C_2_R_2_] (R = ^*t*^Bu, Mes) as building blocks in metallo-supramolecular chemistry was investigated and self-assembly processes with Cu(i) halides resulted in the formation of a large variety of unprecedented one-, two- and even three-dimensional aggregates. The 3D networks showed an interesting topological similarity to allotropes of carbon: diamond and the theoretically proposed polybenzene. Furthermore, the negative charge of the phospholyl ligand favoured the generation of cationic Cu_*a*_X_*b*_ (*a* > *b*, X = Cl, Br, I) assemblies, a challenging area within the well-studied coordination chemistry of CuX units. In addition, the 1D strands were also characterized in solution, revealing the presence of oligomeric units.

## Abstract 2

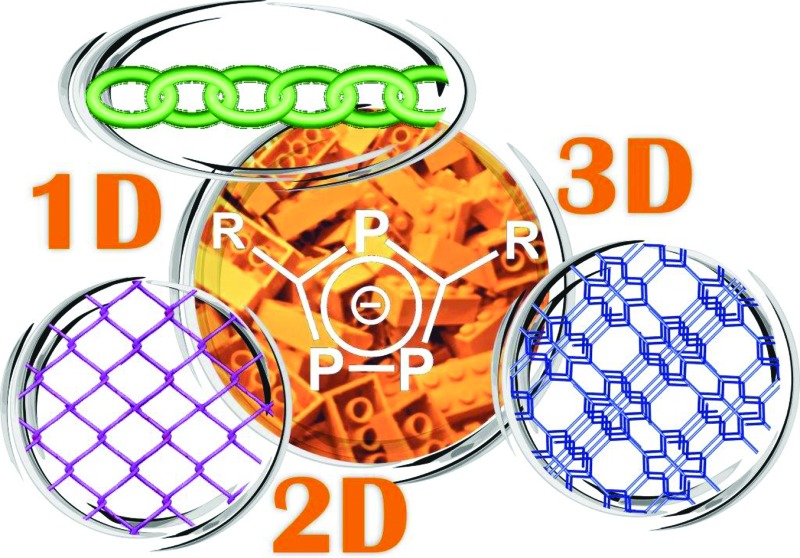
The potential of K[P_3_C_2_R_2_] (R = ^*t*^Bu, Mes) as building blocks in metallo-supramolecular chemistry was investigated and self-assembly processes with Cu(i) halides resulted in the formation of a large variety of unprecedented one-, two- and even three-dimensional aggregates.

## Introduction

**Fig. 1:**
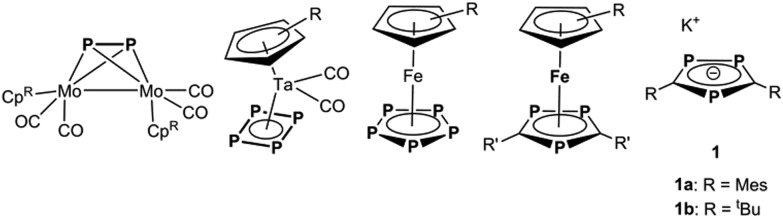
Selected building blocks for metallo-supramolecular chemistry based on phosphorus as donor atoms.

Self-assembly processes and metal–organic frameworks (MOFs) became indispensable principles in supramolecular and coordination chemistry as well as in crystal engineering.^1^ The spontaneous organization of small building blocks to large assemblies by non-covalent interactions is not only of fundamental interest, but also suited for the development of new materials with defined and tunable properties. Particularly, the involvement of coordination bonds in metallo-supramolecular chemistry offers numerous benefits, since they are relatively strong, but often weak enough to show dynamic behaviour. The variety of the used ligands is large; however, the nature of the donor atoms is mostly limited to oxygen, nitrogen or sulphur. So far phosphorus as a donor atom has played only a minor role, opening a field of broad perspectives. A selection of building blocks based on phosphorus as donor atoms is displayed in Fig. 1. In particular, phosphaferrocenes and CuX (X = Cl, Br, I) turned out to be a great combination for the construction of monomeric,^2^ oligomeric,^3^ polymeric^3,4^ and even spherical^5^ coordination compounds. This vast abundance of results can be partially traced back to the variability and flexibility of the coordination behaviour of Cu halides.^6^ Despite this, two aspects still display challenging areas: firstly, though innumerable neutral and anionic aggregates are reported in the literature, cationic Cu_*a*_X_*b*_ (*a* > *b*) assemblies occur only sporadically. Secondly, the formation of 1D strands or 2D networks is well known, though the isolation of 3D assemblies with phosphorus as a donor atom was only possible in very rare cases. To the best of our knowledge, the only examples of 3D aggregates are built up by using inorganic cage molecules^7^ or an organic linker containing a PPh_3_ group.^8^ However, especially in view of future usage such as gas storage and catalytic activities, three-dimensional aggregates seem to be the most promising candidates.

**Fig. 2:**
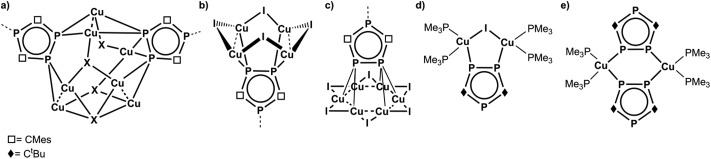
Selected coordination compounds containing triphospholyl ligands and Cu(i) halides.

Recently, we have reported on an unexpected fragmentation of the triphosphaferrocene [Cp*Fe(η^5^-P_3_C_2_Mes_2_)] (Cp* = η^5^-C_5_Me_5_, Mes = 2,4,6-trimethylphenyl) into [Cp*Fe]^+^ and [P_3_C_2_Mes_2_]^–^ moieties, while reacting it with CuX (X = Cl, Br, I).^9^ The remaining phospholyl ligands [P_3_C_2_Mes_2_]^–^ serve as building blocks for a variety of coordination compounds with rare or even novel structural motifs (Fig. 2a–c). Due to the negative charge of this ligand, a buildup of cationic Cu_*a*_X_*b*_ aggregates is predetermined. Since the synthesis of [Cp*Fe(η^5^-P_3_C_2_Mes_2_)] starts from [K(P_3_C_2_Mes_2_)], FeBr_2_ and LiCp*,^10^ the question arises, if the detour of its synthesis and subsequent fragmentation can be avoided. Therefore, we were interested in the use of [K(P_3_C_2_Mes_2_)] itself as a building block. In the literature, the use of 1,2,4-triphospholyl salts [Q(P_3_C_2_R_2_)] (Q = Li, K; R = Mes, ^*t*^Bu, Ph) was primarily made for the preparation of the sandwich complexes tri- and hexaphosphametallocenes^11^ or for coupling reactions resulting in phosphorus rich cage compounds.^12^ Investigations concerning its coordination chemistry towards coinage metal salts are rare and mostly started not from its potassium or lithium salts, but from its neutral trimethylstannyl-triphosphole derivatives.^13^ Only Nixon *et al.* treated [K(P_3_C_2_^*t*^Bu_2_)] with Et_3_PAuCl and Cu_2_I_2_/PMe_3_, respectively, and obtained monomeric (for Cu see: Fig. 2d) or dimeric products (for Cu see: Fig. 2e).^14^ In these reactions the presence of the ligands PEt_3_ and PMe_3_, respectively, impedes further aggregation.

**Scheme 1:**
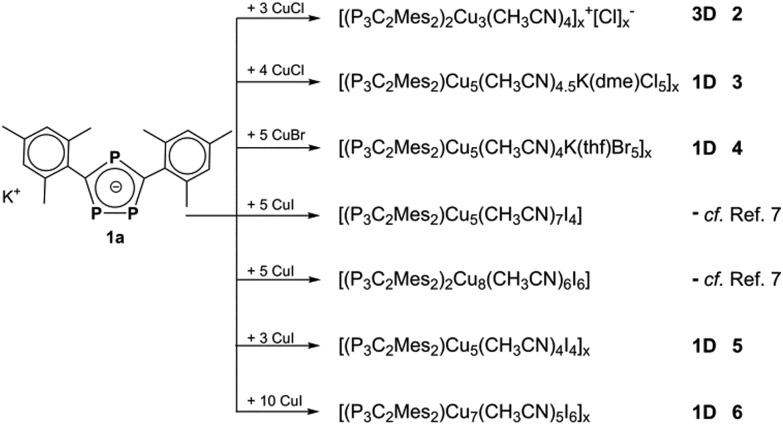
Reactions of **1a** with CuX (X = Cl, Br, I).

Herein, we report on the self-assembly process of [K(P_3_C_2_Mes_2_)] (**1a**) with CuX (X = Cl, Br, I) yielding the monomeric compounds [(μ,η^1^:η^2^:η^2^-P_3_C_2_Mes_2_){Cu(CH_3_CN)(μ_2_-I)}_4_{Cu(CH_3_CN)_3_}] and [(μ,η^1^:η^3^:η^3^-P_3_C_2_Mes_2_)_2_{Cu(CH_3_CN)_3_}_2_{Cu(μ_2_-I)}_6_], the 3D network **2** as well as the 1D polymers **3**, **4**, **5**, **6** (Scheme 1).

**Scheme 2:**
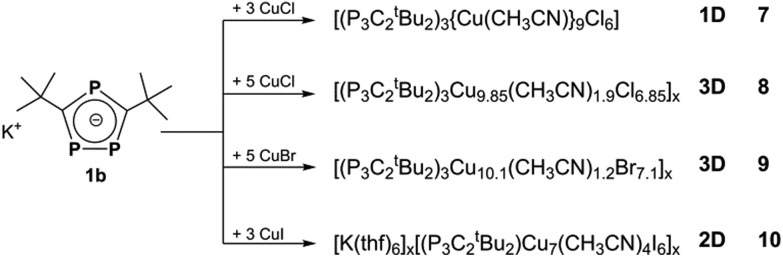
Reactions of **1b** with CuX (X = Cl, Br, I).

Furthermore, by using the ^*t*^Bu derivative [K(P_3_C_2_^*t*^Bu_2_)] (**1b**), 1D polymer **7**, 3D aggregates **8** and **9** and the 2D network of **10** (Scheme 2) could be isolated.

## Results and discussion

Reactions of **1** with CuX (X = Cl, Br, I) were carried out by two different approaches: a yellow to orange solution of **1** in thf or dme can either be layered with a colourless solution of CuX in CH_3_CN or both solutions are stirred together. In all cases, an immediate colour change to deep red can be observed. Depending on the presence and amount of CH_3_CN, concentration, molar ratio, crystallisation conditions and the R group in the phospholyl ligand (R = Mes, ^*t*^Bu), a variety of different products can be isolated (Schemes 1 and 2), even with the same halide. In these cases, the compounds generally crystallize separately and not as mixtures and therefore, a selective synthesis is possible in most instances (for detailed conditions see below and the ESI‡). In doing so, the stoichiometric amount of CuX is the most significant factor for a directed synthesis, as can be seen in Schemes 1 and 2. Furthermore, all crystal structures contain additional solvent molecules, which are discussed only in the ESI‡ in detail. Since several coordination compounds containing **1a** exist due to the fragmentation of [Cp*Fe(η^5^-P_3_C_2_Mes_2_)],^9^ the question arises, if they can also be synthesized using **1a** as the starting material. Indeed, with CuI two known products can be obtained. Both are monomeric compounds, the one with a Cu_4_I_4_ unit forming a crown-like structural motif (Fig. 2b), and the other with a Cu_6_I_6_ six-point star-like arrangement, which is coordinated by one phospholyl ligand from each side (Fig. 2c). But surprisingly, these two represent the only previously observed examples. In fact, a great pool of novel coordination polymers with different structural motifs is obtained and is described henceforth. One common feature among them is the coordination of all three phosphorus atoms of the triphospholyl ring to form polymeric aggregates. Furthermore, CuCl and CuBr tend to form isotypical compounds (*cf.* compounds **3** and **4** and **7** and **8**, respectively), whereas CuI-containing frameworks often differ in their structural motifs. Another determining factor is the substitution pattern in the phospholyl ligand. The sterically demanding Mes ligand almost exclusively leads to the formation of 1D strands with compound **2** as an exceptional case. In contrast, smaller ^*t*^Bu groups allow the aggregation in all directions. In addition, compounds **2–9** show short X···H (X = Cl, Br, I) distances (<Σ_vdW-radii_) with methyl groups of acetonitrile and the R group (R = Mes, *t*Bu) and therefore, weak interactions within the chain or layer as well as between them are indicated (for pictures see the ESI‡). Henceforth, the obtained products are described in relation to their dimensionality.

### One-dimensional polymers (**3–7**)

**Fig. 3:**
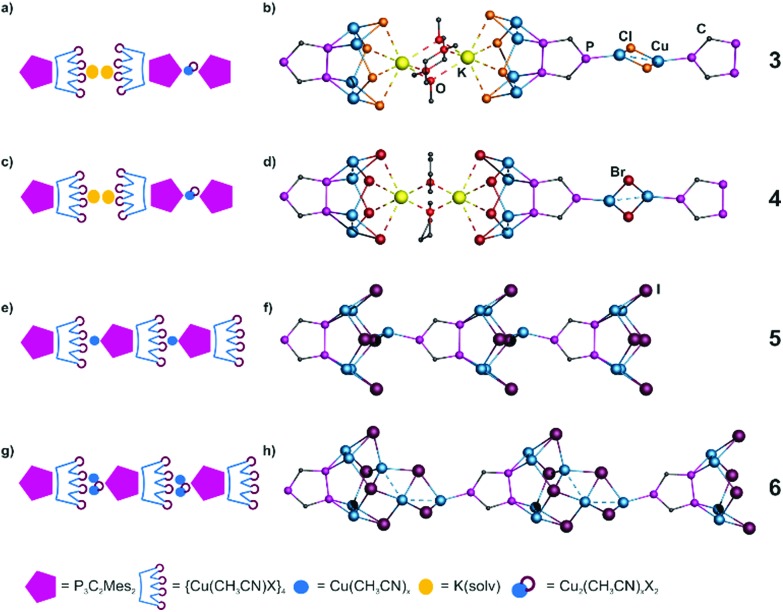
Left: schematic representations of the structures of (a) **3**; (c) **4**; (e) **5** and (g) **6**; right: sections of the polymeric structures of (b) **3**; (d) **4**; (f) **5** and (h) **6**. Mesityl and CH_3_CN ligands, H atoms, solvents and minor positions of disordered groups are omitted for clarity.

The mesityl group in **1a** exhibits a high steric demand, so that an aggregation in one direction is feasible and preferred. Surprisingly, most of the products (**3–6**) show the structural motif of an eight membered Cu_4_X_4_ ring. Its distortion and close Cu···Cu contacts lead to a crown-like arrangement (Fig. 2b and 3, left). This is so far only known for X = I^9,15^ and here it is rather unusual, since (CuI)_4_ units tend to form heterocubanes or ladders.^6^

Hence, **3** and **4** show the first examples for the Cl- and Br-derivatives of this structural motif, respectively. Compound **3** can be isolated as yellow-orange prisms in good yields (57%) and compound **4** as yellow-orange blocks in very good yields (88%). Compounds **3** and **4** both crystallize as solvates in monoclinic space groups (**3**: *P*2_1_/*n*, **4**: *C*2/*m*) and display isotypical compounds (Fig. 3a–d). The Cu_2_-dimers in the formed crown show distances of 2.545(1) and 2.553(1) Å for **3** and 2.548(1) Å for **4**. They are coordinated by the adjacent P atoms of **1a** with bond lengths of 2.274(1)–2.296(1) Å in **3** and 2.280(1)–2.294(1) Å in **4**. The halides additionally interact with a potassium centered above the ‘crown’ (K···Cl: 3.080(2)–3.179(2) Å; K···Br: 3.210(1)–3.344(1) Å). The coordination sphere is completed by two additional thf (in **3**) or dme (in **4**) molecules. These solvent ligands exhibit a bridging coordination mode and therefore connect two [(P_3_C_2_Mes_2_)(Cu_4_X_4_)] units. The aggregation is accomplished by a Cu_2_X_2_ four-membered ring, which is coordinated by isolated P atoms of **1a** from each side. These four-membered rings are severely disordered over two (in **3**) up to eight (in **4**) positions around the direction of the chain and in some positions the Cu atoms are additionally coordinated by CH_3_CN, therefore, some of them show a trigonal, and some a tetrahedral environment (see the ESI‡).

Using CuI, two slightly different 1D polymers (**5**, **6**) were isolated (Fig. 3e–h). Compound **5** crystallizes as red-brown blocks in the tetragonal space group *P*4/*n*, and compound **6** as orange lath-shaped crystals in the triclinic space group *P*1. The Cu_4_I_4_ arrangement is similar to **3** and **4** with close Cu···Cu distances (**5**: 2.541(4) Å; **6**: 2.523(2) Å) and Cu–P bond lengths (**5**: 2.277(5)–2.319(6) Å; **6**: 2.285(2) Å–2.319(2) Å). In **5**, this unit is polymerized into a 1D chain by one additional Cu, coordinated by the isolated P atom of **1a** (P–Cu 2.249(6) Å) and two I-tips of the ‘crown’ (Fig. 3e and f).

The diffraction pattern of **5** shows quite strong diffuse scattering (see the ESI‡) caused by correlated disorder of the {Cu_5_I_4_(CH_3_CN)_4_(P_3_C_2_Mes_2_)} repeating units within the polymeric chain. Our attempts to model this effect allowed us to assume that there is a strong negative correlation (alternation of repeating units’ rotations) within the polymeric chains together with the weaker negative correlation between neighboring chains (see the ESI‡ for details).

In contrast, in **6** a Cu_3_I_2_ fragment serves as a linkage between the top of the crown and the third P atom of the phospholyl ligand (P–Cu 2.214(2) Å) (Fig. 3g and h). The formation of **5** and **6** is remarkable, since the reaction of [Cp*Fe(η^5^-P_3_C_2_Mes_2_)] and CuI also gives a 1D polymer containing the same structural motif, but polymerized *via* a Cu_3_I_2_ five-membered ring. So, these results again demonstrate the structural variability of the Cu(i) halides, especially of CuI. The orientation of **1a** in **5** and **6** is the same along the chain, while in **3** and **4** they show an alternating orientation. Note that the Cu_*a*_I_*b*_ assembly is positively charged in both polymers, namely [Cu_5_I_4_]^+^ in **5** and [Cu_7_I_6_]^+^ in **6**. Thus, these examples expand the small and unexplored area of cationic Cu_*a*_X_*b*_ clusters. The reason for their formation can most probably be traced back to the use of the negatively charged triphospholyl ligand. This approach was also used in two other examples, in which anionic triazolate and tetrazolate linking units were used, respectively.^16^

Summing up the results of the reaction of **1a** with CuI, four different coordination compounds are obtained. Fortunately, a selective synthesis can be controlled mainly by stoichiometry. For example, an excess of copper iodide (**1a** : Cu = 1 : 10) leads to the crystallization of compound **6** solely, which is in agreement with the highest molar ratio of Cu in **6** (**1a** : Cu = 1 : 7). In contrast, the monomeric compound with the Cu_6_ star-like arrangement (Fig. 2c) contains the lowest molar ratio of Cu (**1a** : Cu = 1 : 4) and can therefore be obtained, when less equivalents of CuI are used. However, not all attempts to reproduce polymer **5** were successful, most likely due to the preferred crystallization of its monomeric derivative (Fig. 2b) with the exact same molar ratio (**1a** : Cu = 1 : 5). Also the variation in the concentration and solvent was not successful in all of the attempts.

**Fig. 4:**
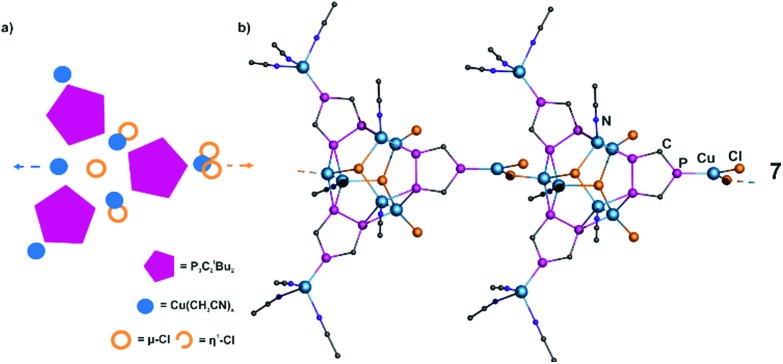
(a) Schematic representation of the structure of **7**. (b) Section of the polymeric structure of **7**. ^*t*^Bu ligands, H atoms, solvents and minor positions of disordered CH_3_CN are omitted for clarity.

The 1D polymer **7** crystallizes as orange plates in the monoclinic space group *P*2_1_/*m* and reveals a structural motif different from the other 1D polymers, since it contains **1b** as a building block and it also differs from the general structural motif (Fig. 4). Due to the lower steric demand of the ^*t*^Bu group, the arrangement of three phospholyl rings close to each other is possible. They are connected by three Cu_2_-dimers (Cu···Cu 2.482(1)–2.496(1) Å), which form a triangular prism (P–Cu 2.325(1)–2.361(1) Å). The prism is capped by two μ_3_-Cl with μ_3_-Cl–Cu 2.390(1)–2.441(1) Å bond lengths. Each Cu atom additionally coordinates either Cl^–^ or CH_3_CN moieties to reach a tetrahedral environment. Two of these three halides are terminals (η^1^-Cl–Cu 2.231(1) Å), while the third coordinates another copper atom (μ_2_-Cl–Cu 2.279(2)–2.300(2) Å), which is in turn bound to an isolated P atom of **1b**. The remaining P atoms of the other two ligands prevent polymerization in other directions by coordination of terminal Cu(CH_3_CN)_3_ units (Cu–P 2.191(2) Å). Therefore, compound **7** displays a 1D polymer. The central assembly with **7** Cu^+^ and **6** Cl^–^ can be regarded as an isomeric unit to the assembly in **6** and is another unprecedented example of a cationic Cu_*a*_X_*b*_ unit.

### Two-dimensional assembly (**10**)

**Fig. 5:**
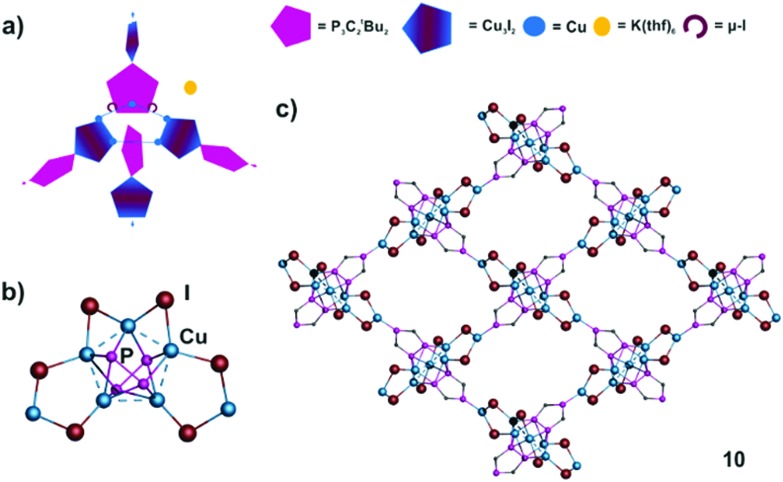
(a) Schematic representation of the structure of **10**. (b) Core motif of **10**. (c) Section of the 4-connected anionic polymer network of **10**. ^*t*^Bu and CH_3_CN ligands, H atoms, solvents and counterions are omitted for clarity.

Starting from **1b** and CuI as building blocks occasionally the 2D network **10** can be isolated. Compound **10** crystallizes as dark red brown blocks in the orthorhombic space group *Pbcn*. In **10**, a central Cu_5_-ring with Cu···Cu distances in the range from 2.540(4) to 2.796(4) Å is coordinated from each side by two units of **1b**, which are perpendicular to each other (Cu–P 2.256(5)–2.564(5) Å) (Fig. 5a and b). Four Cu atoms are parts of two Cu_3_I_2_-rings, a building unit, which also occurs in **8** and **9**. The coordination sphere of the remaining Cu is saturated by two μ_2_-I ligands (Cu–I 2.483(3)–2.650(3) Å), and hence a tetrahedral environment results for each copper atom. Aggregation takes place by the coordination of the isolated P atom of **1b** to the isolated Cu atom of the perpendicular Cu_3_I_2_ ring *via* a relatively short Cu–P bond of 2.215(5) Å. Through the propagation in four directions within the layer a mesh-like structure is formed (Fig. 5c). Furthermore, **10** also displays an unprecedented representative for a cationic copper halide aggregate, since **7** Cu^+^ and **6** I^–^ are present in the repeating unit. In total, the combination with two units of **1b** even leads to an anionic assembly, which is balanced by K(thf)_6_^+^ cations embedded in the mashes and separating the layers from each other (for pictures see ESI‡). Together with the ^*t*^Bu groups of **1b** the meshes do not provide free space. Due to the alternating arrangement of cationic and anionic ‘layers’ no short I···H distances can be found in the crystal structure of **10**.

### Three-dimensional networks (**2**, **8**, **9**)

Surprisingly, from the reaction of **1a** with CuCl as a second product also the 3D polymer **2** can be isolated, despite the sterically demanding Mes group. Since they differ significantly in their Cu content, a directed synthesis is enabled by using different stoichiometries. More than three equivalents of CuCl lead to the formation of **3** (**1a** : Cu = 1 : 5), whereas three or less equivalents of CuCl result in the crystallisation of **2** (**1a** : Cu = 1 : 1.5).

**Fig. 6:**
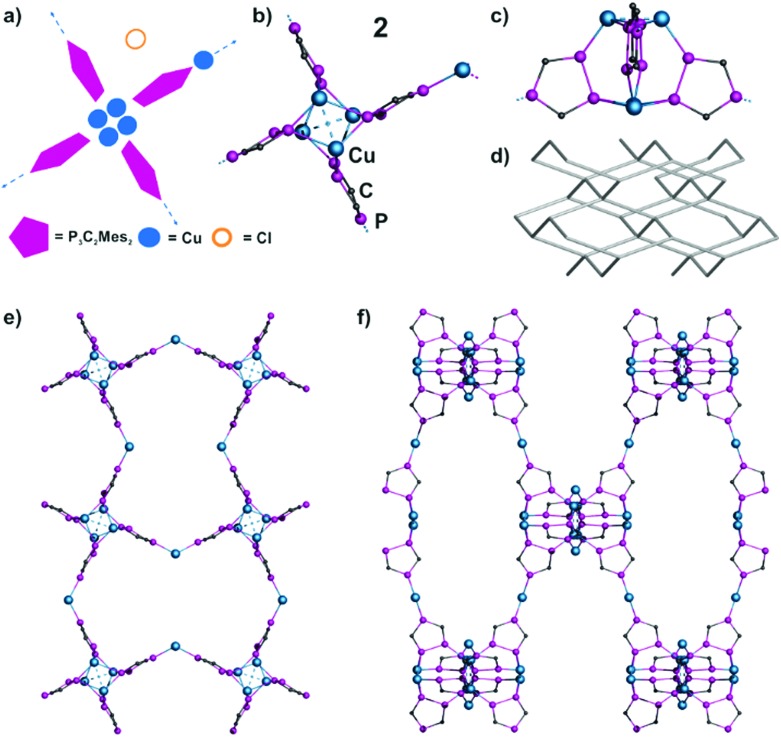
(a) Schematic representation of the structure of **2**. (b) Central structural motif of **2** (top view). (c) Central motif of **2** (lateral view) illustrating the tetrahedral nodes. (d) Fragment of the **dia** net. (e)–(f) Sections of the cationic polymer network of **2**. View along the (e) *c*-axis, and (f) *a*-axis. Mes and CH_3_CN ligands, H atoms, counterions and solvents are omitted for clarity.

Compound **2** crystallizes as red blocks in the tetragonal space group *I*42*d* (see also the ESI‡). The repeating unit contains four phospholyl ligands **1a**, whose adjacent P atoms are connected *via* two Cu_2_ dimers (Cu···Cu 2.555(2) Å; P–Cu 2.181(4)–2.503(5) Å) (Fig. 6a–c). Hence, this is the only assembly, whose central core structure does not include a halide and is built up only by **1a** and Cu units. The linkage of the remaining P atoms of the phospholyl rings by Cu(CH_3_CN)_2_ leads to a polymeric structure. Charge balance is afforded by the presence of an uncoordinated Cl^–^ per repeating unit (4 **1a** ^–^, 5 Cu^+^, 1 Cl^–^). In addition, this core acts as a tetrahedral node and induces a propagation in the other two dimensions, resulting in a 3D network (Fig. 6e–f). Its net topology can be assigned to the **dia** type (Fig. 6d).^17^ Hence, it shows topological similarity with diamond, which gives this net its name.

**Fig. 7:**
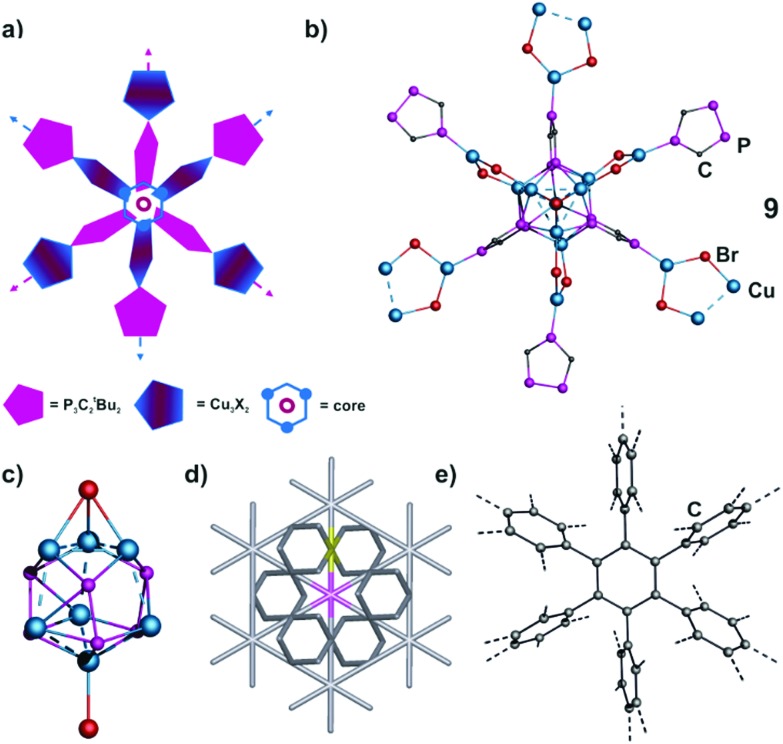
(a) Schematic representation of the structures of **8** and **9**. (b) Section of the polymeric structure of **9**. ^*t*^Bu and CH_3_CN ligands, H atoms, solvents and minor positions of disordered fragments are omitted for clarity. (c) Major part of the central core motif in **9** (87%). (d) Fragment of the **hxg** net with mutually tilted 6-connected nodes marked in magenta and yellow. (e) Section of the polymeric structure of polybenzene (simulated).

Also with R = ^*t*^Bu in **1b** the formation of three dimensional networks is observed. When a solution of **1b** in dme is layered with a solution of CuX (X = Cl, Br), at the phase boundary the formation of big dark red blocks of **8** (X = Cl) and **9** (X = Br), respectively, can be readily observed after one day. Both compounds are isotypical and crystallize in the cubic space group *F*43*c.* However, their core structures turned out to be severely disordered. The crystal structure of **9** is more ordered (see the ESI‡ for details), and therefore, it is described first. The central structural motif of **9** contains three Cu_2_-dimers and the adjacent P atoms of three units of **1b**, which are arranged according to a distorted hexagonal P_6_Cu_6_ prism (Cu···Cu 2.694(1)–2.783(2) Å; Cu–P 2.298(2)–2.620(2) Å) (Fig. 7a–c). This prism is capped by copper on one side and by a CuBr unit on the other side. In addition, each Cu_2_-dimer is a component of a Cu_3_Br_2_ five-membered ring (similar to **10**). This description is the case for 87% of **9**, whereas in its minor part (13%) the μ_3_-Br coordinates an additional Cu(CH_3_CN)_3_ unit, and some Br positions are replaced by acetonitrile ligands (*cf*. ESI‡). In **8**, the disorder is similar and the major part remains the same as in **9**. However, different occupation factors and additional Cu deficiencies make its description more complicated and are explained in detail in the ESI.‡ In total, the differences in the occupation factors of the disordered fragments for **8** and **9** and the requirement of a charge balance lead to the sum formulae [(P_3_C_2_^*t*^Bu_2_)_3_Cu_9.85_Cl_6.85_(CH_3_CN)_1.9_]_*x*_ for **8** and [(P_3_C_2_^*t*^Bu_2_)_3_Cu_10.1_Br_7.1_(CH_3_CN)_1.2_]_*x*_ for **9**.

Interestingly, the core of both compounds **8** and **9** is proceeded to be stellar-like to give a 6-connected 3D network (Fig. 7a and b). It demonstrates the topological similarity with one of the theoretically possible allotropes of carbon, so-called polybenzene or cubic graphite (Fig. 7e). The polybenzene structure can be derived from a benzene molecule if every hydrogen atom is replaced by a phenyl ring, in which every hydrogen atom is in turn replaced by a phenyl ring, *etc.* (Fig. 7e).^18,19^ This structure was first predicted by Gibson *et al.* in 1946.^20^ The calculations based on first-principles molecular dynamics showed that this carbon allotrope should be quite stable, but so far no synthetic approach has been found.^19^

If one treats the phenyl ring as a ‘node’ of the framework, the resulting 6-connected 3D net belongs to the **hxg** topological type (Fig. 7d),^21^ the same as the found 3D framework in **8** and **9** irrespective of any disorder. Eight other crystal structures of coordination polymers retain the same topological type (see ESI‡). In these complexes the {M_3_(μ_3_-O)}^4+^ or {M_3_(μ_3_-OH)}^5+^ unit (M^2+^ = Cu^2+^, Ni^2+^) joins to each other by using N-heterocyclic bridging ligands like 1,2,4-triazolate,^22^ 5-(4-pyridyl)tetrazolate,^23^ or pyrazole-4-carboxylate.^24^ Interestingly, all structures crystallize or are described in cubic space groups, *Fd*3*c* ({M_3_(μ_3_-O)}- or {M_3_(μ_3_-OH)}-based polymers), *Pn*3*m* (polybenzene) and *F*43*c* (**8** and **9**).

Whereas crystals of **2** decomposed rapidly in air, the crystals of **8** and **9** turned out to be extremely stable. They were kept in air for two years, intriguingly without losing crystallinity. A repeated elemental analysis of **9** and X-ray diffraction experiments of **8** and **9**, respectively, proved the identical composition and excluded oxidation.

Since the combination of CuCl and **1b** leads to two different compounds (**7** and **8**), the conditions of a directed synthesis were investigated. In this case, the used method turned out to be of importance. Since **7** is soluble, it can be isolated by stirring experiments, while diffusion methods exclusively lead to the crystallization of the completely insoluble polymer **8** at the phase boundary. As a supporting factor, a higher molar ratio is used for the synthesis of **8** (**1b** : Cu = 1 : 5).

### Characterization in solution

All received compounds are insoluble in common solvents like hexane, toluene, Et_2_O, thf, dme and CH_2_Cl_2_. Only the 1D polymers (**3–7**) are soluble in CH_3_CN to give yellow to deep red solutions. If a coordination to Cu (nuclear spin *I* = 3/2) is still present in solution, one would expect two broad signals in the corresponding ^31^P{^1^H} NMR spectra. This is the case for **6** and **7**. The signals of **6** appear at *δ* = 136 and 222 ppm with the integral ratio of 2 : 1 for the adjacent and the isolated P atoms of the phospholyl ring, respectively. In comparison with the salt **1a** (*δ* = 261.7 ppm (t, 1P) and 266.4 ppm (d, 2P)), both signals are shifted to higher field. Interestingly, the adjacent phosphorus atoms show a much more intensive shift (130 ppm) than the isolated one (40 ppm), so that the order of the signals is inverted. In contrast, the signals of the adjacent P atoms of **1b** (*δ* = 246.3 ppm (d)) are shifted to higher field than the isolated one (*δ* = 254.9 ppm (t)), which is reversed in comparison with **1a**. However, in **7** this trend does not occur and the signals of **7** (*δ* = 137 (2P) and 264 (1P) ppm) appear in the same order as in the spectra of **1a** and **6**. Hence, the signal of the isolated phosphorus shows a slight downfield shift of 11 ppm, whereas the signal of the adjacent P atoms is strongly upfield shifted by 109 ppm.

In contrast, the ^31^P{^1^H} NMR spectrum of **3** shows only one small broad signal at 205 ppm, so Cu remains coordinated to the phospholyl ligand in solution. The lack of a second signal might be a hint of the equivalence of the P atoms. However, a more likely reason is the disappearance of the other signal below the noise floor due to its broadness, since the quality of the spectrum is due to the bad solubility already quite poor.

Surprisingly, the ^31^P{^1^H} NMR spectrum of the isotypical compound **4** shows three signals. Due to the identical shift the signal at *δ* = 138 ppm can be assigned to the isolated P atom of the phospholyl ligand. The other two signals appear at 204 and 217 ppm, respectively. This indicates the presence of two different species in the ratio of 3 : 1. The signals might be attributed to a smaller, monomeric and a larger, oligomeric unit, as it has been observed for other phospholyl-based polymers.^9^

The size of the aggregate in solution cannot be derived from the NMR spectra, though hints for at least oligomeric units are also given by mass spectrometry. The corresponding cationic ESI mass spectra show fragments up to [(P_3_C_2_Mes_2_)_4_Cu_11_Cl_6_]^+^, [(P_3_C_2_Mes_2_)_4_Cu_10_Br_5_]^+^, [(P_3_C_2_Mes_2_)_4_Cu_10_I_5_]^+^ and [(P_3_C_2_^*t*^Bu_2_)_7_Cu_16_Cl_8_]^+^, respectively.

The 2D and 3D networks are insoluble even in CH_3_CN. However, its analysis by ESI mass spectrometry was able for **2** and **9** after sonication, which leads to a degradation of the 3D network. The corresponding spectra look almost the same as the above mentioned ones with [(P_3_C_2_Mes_2_)_5_Cu_8_Cl_2_]^+^ as the biggest fragment for **2** and [(P_3_C_2_^*t*^Bu_2_)_7_Cu_17_Br_9_]^+^ for **9**.

Furthermore, solutions of the polymeric compounds **2**, **4**, **7–9** as well as the monomeric assembly [(P_3_C_2_Mes_2_){Cu(CH_3_CN)(μ_2_-I)}_4_{Cu(CH_3_CN)_3_}] in CH_3_CN were analysed by UV-vis spectroscopy, revealing partially overlapping absorption bands (for details and spectra see the ESI‡). However, a dependency of the dimensionality of the network or the halide cannot be deduced. In contrast, a relationship between the R group is indicated, since all solutions of the mesityl derivatives are red in colour with an absorption band at *λ* = 532 nm, whereas the ^*t*^Bu substituted assemblies give yellow solutions. Moreover, these results are in agreement with the above discussed depolymerisation behaviour of the products in donor solvents like CH_3_CN.

## Conclusions

In summary, the triphospholyl ligands **1a** and **1b** were introduced as building blocks in supramolecular chemistry. The self-assembly processes with CuX (X = Cl, Br, I) led to the formation of unprecedented polymeric networks. The negative charge of the cyclic ligand favoured the aggregation of cationic Cu_*a*_X_*b*_ (*a* > *b*) assemblies, which so far have been only rarely observed. The 1D strands in **3–6** show a rather uncommon crown-like arrangement. The 2D network of **10** is comparable to a wire-mesh. Even the selective synthesis of three dimensional aggregates is possible, all of them with an interesting structure. The net of **2** reveals tetrahedral nodes and a resulting **dia**-topology and is therefore related to diamond. Furthermore, the star-like build-up of compounds **8** and **9** can be assigned to the **hxg**-topology and hence, it shows structural analogy to ‘polybenzene’. This allotrope of carbon is proposed to be quite stable, however, has not been synthesized so far. The results nicely demonstrate the potential of the triphospholyl ligands in supramolecular chemistry, especially for the formation of MOF-like assemblies.
