# Supplementary material for: 1,2,4-Triphospholyl anions – versatile building blocks for the formation of 1D, 2D and 3D assemblies
Source: Dalton Trans. 2015 May 11;44(22):10245–52. doi: 10.1039/c5dt01230a (PMC4447064; doi:10.1039/c5dt01230a)
Supplement: Supplementary file 1 [file DT-044-C5DT01230A-s001.pdf]

Electronic Supplementary Material (ESI) for Dalton Transactions.
This journal is © The Royal Society of Chemistry 2015

Supplementary information for the manuscript

1,2,4-Triphospholyl Anions – Versatile Building Blocks for the Formation of 1D, 2D and 3D Assemblies

C. Heindl, Eugenia V. Peresypkina, Alexander V. Virovets, Vladislav Yu. Komarov and M. Scheer*

List of numbered compounds:
For co-crystallized solvent molecules see X-ray part.

1a $[K(P_3C_2Mes_2)]$

1b $[K(P_3C_2{}^tBu_2)]$

2 $[(\mu,\eta^1:\eta^1:\eta^2\text{-}P_3C_2Mes_2)_2\{Cu(CH_3CN)\}_2\{Cu(CH_3CN)_2\}]^+{}_x[Cl^-]_x$

3 $[(\mu,\eta^1:\eta^2:\eta^2\text{-}P_3C_2Mes_2)\{Cu(CH_3CN)(\mu_2\text{-}Cl)\}_4\{K(\mu_2\text{-dme})\}\{Cu(CH_3CN)_{0.5}(\mu_2\text{-}Cl)\}]_x$

4 $[(\mu,\eta^1:\eta^2:\eta^2\text{-}P_3C_2Mes_2)\{Cu(CH_3CN)(\mu_2\text{-}Br)\}_4\{K(\mu_2\text{-thf})\}\{Cu(\mu_2\text{-}Br)\}]_x$

5 $[(\mu,\eta^1:\eta^2:\eta^2\text{-}P_3C_2Mes_2)\{Cu(CH_3CN)(\mu_2\text{-}I)\}_2\{Cu(CH_3CN)(\mu_3\text{-}I)\}_2Cu]_x$

6 $[(\mu,\eta^1:\eta^2:\eta^2\text{-}P_3C_2Mes_2)\{Cu(CH_3CN)(\mu_3\text{-}I)\}_2\{Cu(CH_3CN)(\mu_4\text{-}I)\}_2Cu_2\{Cu(CH_3CN)\}(\mu_3\text{-}I)(\mu_2\text{-}I)]_x$

7 $[(\mu,\eta^1:\eta^2:\eta^2\text{-}P_3C_2tBu_2)(CuCl)_3(\mu_3\text{-}Cl)_2\{Cu(CH_3CN)\}_3\{Cu(\mu_2\text{-}Cl)\}\{Cu(CH_3CN)_3\}_2]_x$

8 $[(P_3C_2tBu_2)_3Cu_{9.85}Cl_{6.85}(CH_3CN)_{1.9}]_x$

9 $[(P_3C_2tBu_2)_3Cu_{10.1}Br_{7.1}(CH_3CN)_{1.2}]_x$

10 $[K(thf)_6]_x[(\mu,\eta^1:\eta^3:\eta^3\text{-}P_3C_2tBu_2)_2\{Cu_3(\mu_2\text{-}I)_2\}_2\{Cu(\mu_2\text{-}I)_2\}]_x$

Content:

1. Experimental Part 2

2. X-ray Structure Analysis 7

3. Crystallographic problem in 2: Quality of the diffraction experiment 24

4. Diffuse scattering in 5 29

5. Topological analysis of the 3D polymers 32

6. Pictures of the crystal packing of the polymers 2 - 10 32

7. UV-vis Spectroscopy 36

1. Experimental Part

All reactions were performed under an inert atmosphere of dry nitrogen or argon with standard vacuum, Schlenk, and glove-box techniques. Solvents were purified, dried and degassed prior to use by standard procedures. $[K(P_3C_2Mes_2)]$[1] and $[K(P_3C_2{}^tBu_2)]$[2] were synthesized following reported procedures. Commercially available chemicals were used without further purification. Solution NMR spectra were recorded on either Bruker Avance 300 or 400 spectrometer. The corresponding ESI-MS spectra were acquired on a ThermoQuest Finnigan MAT TSQ 7000 mass spectrometer, while elemental analyses were performed on a Vario EL III apparatus.

1.1 Synthesis of 2

In a Schlenk tube a solution of $[K(P_3C_2Mes_2)]$ (66 mg, 0.17 mmol) in dme (10 mL) is layered with a solution of CuCl (50 mg, 0.5 mmol) in CH_3CN (10 mL). At the phase boundary a color change to deep red can be observed. After complete diffusion and precipitation of a beige powder the red solution is decanted. While storing at 8°C the formation of red rods of **2** can be observed within a few days. The mother liquor is decanted, the crystals are washed with hexane (3 x 5 mL) and dried in vacuo. By concentrating the mother liquor and layering with Et_2O, a second crop of crystals can be obtained.

Analytical data of **2**:

Yield: 40 mg (0.34 mmol, 20%)

^{1}H NMR (CD_3CN): δ [ppm] = 2.02 (s, 3H, *o*-CH_3), 2.11 (s, 3H, *o*-CH_3), 2.25 (s, 3H, *p*-CH_3), 3.28 (s, $(C\underline{H}_3OCH_2)_2$), 3.45 (s, $(CH_3OC\underline{H}_2)_2$), 6.88 (s, 2H, *aryl*-H).

Positive ion ESI-MS (CH_3CN): *m/z* (%) = 2356.7 $[\{P_3C_2Mes_2\}_5Cu_8Cl_2]^+$, 2257.6 $[\{P_3C_2Mes_2\}_5Cu_7Cl]^+$, 2036.4 $[\{P_3C_2Mes_2\}_4Cu_8Cl_3]^+$, 1936.6 $[\{P_3C_2Mes_2\}_4Cu_7Cl_2]^+$, 1837.6 $[\{P_3C_2Mes_2\}_4Cu_6Cl]^+$, 1739.6 $[\{P_3C_2Mes_2\}_4Cu_5]^+$, 1517.3 $[\{P_3C_2Mes_2\}_3Cu_6Cl_2]^+$, 1419.2 $[\{P_3C_2Mes_2\}_3Cu_5Cl]^+$, 1319.3 $[\{P_3C_2Mes_2\}_3Cu_4]^+$, 998.9 $[\{P_3C_2Mes_2\}_2Cu_4Cl]^+$, 901.2 $[\{P_3C_2Mes_2\}_4Cu_6]^{2+}$

Negative ion ESI-MS (CH_3CN): *m/z* (%) = 232.6 $[Cu_2Cl_3]^-$, 134.8 (100) $[CuCl_2]^-$

1.2 Synthesis of 3

In a Schlenk tube a solution of $[K(P_3C_2Mes_2)]$ (50 mg, 0.12 mmol) in dme (9 mL) is first layered with a solvent mixture of dme and CH_3CN (1:1; 3 mL), afterwards with a solution of CuCl (51 mg, 0.52 mmol) in CH_3CN (12 mL). After complete diffusion and precipitation of a beige powder the red solution is filtered and concentrated to 5 mL. While storing at -28°C the formation of yellow prisms of **3** can be observed within a few days. The mother liquor is decanted, the crystals are washed with hexane (3 x 5 mL) and dried in vacuo. By concentrating the mother liquor, a second crop of crystals can be obtained.

Analytical data of **3**:

Yield: 66 mg (0.068 mmol, 57%)

^{1}H NMR (CD_3CN): δ [ppm] = 1.95 (s, CH_3CN), 2.03 (s, 3H, *o*-CH_3), 2.19 (s, 3H, *o*-CH_3), 2.26 (s, 3H, *p*-CH_3), 6.90 (s, 2H, *aryl*-H).

^{31}P{^{1}H} NMR (mother liquor, dme/CH_3CN/C_6D_6): δ [ppm] = 31.3 (br), 117.7 (br), 218.3 (br).

Positive ion ESI-MS (CH_3CN): *m/z* (%) = 2333.9 $[\{P_3C_2Mes_2\}_4Cu_{11}Cl_6]^+$, 2232.7 $[\{P_3C_2Mes_2\}_4Cu_{10}Cl_5]^+$, 2134.4 $[\{P_3C_2Mes_2\}_4Cu_9Cl_4]^+$, 2035.2 $[\{P_3C_2Mes_2\}_4Cu_8Cl_3]^+$, 1978.5 $[\{P_3C_2Mes_2\}_4Cu_7Cl_2\{CH_3CN\}]^+$, 1937.1 $[\{P_3C_2Mes_2\}_4Cu_7Cl_2]^+$, 1837.2 $[\{P_3C_2Mes_2\}_4Cu_6Cl]^+$, 1738.5 $[\{P_3C_2Mes_2\}_4Cu_5]^+$, 1617.1 $[\{P_3C_2Mes_2\}_3Cu_7Cl_3]^+$, 1419.4 $[\{P_3C_2Mes_2\}_3Cu_5Cl]^+$, 1319.0 $[\{P_3C_2Mes_2\}_3Cu_4]^+$, 1198.8 $[\{P_3C_2Mes_2\}_2Cu_6Cl_3]^+$, 1139.8 $[\{P_3C_2Mes_2\}_2Cu_5Cl_2\{CH_3CN\}]^+$, 1098.8 $[\{P_3C_2Mes_2\}_2Cu_5Cl_2]^+$, 991.7 $[\{P_3C_2Mes_2\}_4Cu_7Cl\{CH_3CN\}_2]^{2+}$, 970.7 $[\{P_3C_2Mes_2\}_4Cu_7Cl\{CH_3CN\}]^{2+}$, 950.7 $[\{P_3C_2Mes_2\}_4Cu_7Cl]^{2+}$, 901.2 $[\{P_3C_2Mes_2\}_4Cu_6]^{2+}$

Negative ion ESI-MS (CH_3CN): *m/z* (%) = 530.4 $[Cu_5Cl_6]^-$, 232.8 $[Cu_2Cl_3]^-$, 135.0 (100) $[CuCl_2]^-$

Elemental analysis: Calculated (%) for $[(P_3C_2Mes_2)Cl_5Cu_5K(CH_3CN)(dme)_{0.5}]$ (976 g/mol): C 29.55, H 3.10, N 1.44; found: C 29.12, H 3.29, N 1.83.

1.3 Synthesis of 4

In a Schlenk tube a solution of $[K(P_3C_2Mes_2)]$ (50 mg, 0.12 mmol) in dme (9 mL) is first layered with a solvent mixture of dme and CH_3CN (1:1; 3 mL), afterwards with a solution of CuBr (93 mg, 0.52 mmol) in CH_3CN (12 mL). After complete diffusion and precipitation of a beige powder the red solution is stored at -28°C. Within one day the formation of yellow-orange blocks of **4** can be observed. The mother liquor is decanted, the crystals are washed with hexane (3 x 5 mL) and thf (2 x 8 mL) and dried in vacuo. By concentrating the mother liquor and storing it at -28°C, a second crop of crystals can be obtained. **4** can also be synthesized by using thf instead of dme, however due to the lower solubility of $[K(P_3C_2Mes_2)]$ in thf a larger volume is needed (20 mL).

Analytical data of **4**:

Yield: 145 mg (0.106 mmol, 88%)

^{1}H NMR (CD_3CN): δ [ppm] = 1.79 (m, thf), 1.95 (s, CH_3CN), 2.04 (s, 3H, *o*-CH_3), 2.11 (s, 3H, *o*-CH_3), 2.26 (s, 3H, *p*-CH_3), 3.63 (m, thf) 6.89 (s, 2H, *aryl*-H).

$^{31}P\{^1H\}$ NMR (CD_3CN): δ [ppm] = 137.9 (br), 204.3 (br), 216.8 (br).

Positive ion ESI-MS (CH_3CN): *m/z* (%) = 2457.8 $[\{P_3C_2Mes_2\}_4Cu_{10}Br_5]^+$, 2313.5 $[\{P_3C_2Mes_2\}_4Cu_9Br_4]^+$, 2169.1 $[\{P_3C_2Mes_2\}_4Cu_8Br_3]^+$, 2025.2 $[\{P_3C_2Mes_2\}_4Cu_7Br_2]^+$, 1881.4 $[\{P_3C_2Mes_2\}_4Cu_6Br_1]^+$, 1749.0 $[\{P_3C_2Mes_2\}_3Cu_7Br_3]^+$, 1738.6 $[\{P_3C_2Mes_2\}_4Cu_5]^+$, 1474.5 $[\{P_3C_2Mes_2\}_4Cu_{14}Br_8]^{2+}$, 1423.6 $[\{P_3C_2Mes_2\}_4Cu_{13}Br_7\{CH_3CN\}]^{2+}$, 1373.9 $[\{P_3C_2Mes_2\}_4Cu_{12}Br_6\{CH_3CN\}_2]^{2+}$, 1302.0 $[\{P_3C_2Mes_2\}_4Cu_{11}Br_5\{CH_3CN\}_2]^{2+}$, 1280 $[\{P_3C_2Mes_2\}_4Cu_{11}Br_5\{CH_3CN\}]^{2+}$, 1259 $[\{P_3C_2Mes_2\}_4Cu_{11}Br_5]^{2+}$, 1229.5 $[\{P_3C_2Mes_2\}_4Cu_{10}Br_4\{CH_3CN\}_2]^{2+}$, 1208 $[\{P_3C_2Mes_2\}_4Cu_{10}Br_4\{CH_3CN\}]^{2+}$, 1187.6 $[\{P_3C_2Mes_2\}_4Cu_{10}Br_4]^{2+}$, 1156.8 $[\{P_3C_2Mes_2\}_4Cu_9Br_3\{CH_3CN\}_2]^{2+}$, 1107.1 $[\{P_3C_2Mes_2\}_4Cu_8Br_2\{CH_3CN\}_3]^{2+}$, 1086.1 $[\{P_3C_2Mes_2\}_4Cu_8Br_2\{CH_3CN\}_2]^{2+}$, 1063.4 $[\{P_3C_2Mes_2\}_4Cu_8Br_2\{CH_3CN\}]^{2+}$, 1013.7 $[\{P_3C_2Mes_2\}_4Cu_7Br\{CH_3CN\}_2]^{2+}$, 993.4 $[\{P_3C_2Mes_2\}_4Cu_7Br\{CH_3CN\}]^{2+}$, 973 $[\{P_3C_2Mes_2\}_4Cu_7Br]^{2+}$

Negative ion ESI-MS (CH_3CN): *m/z* (%) = 796.2 $[Cu_5Br_6]^-$, 654.5 $[Cu_4Br_5]^-$, 508.5 $[Cu_3Br_4]^-$, 366.7 $[Cu_2Br_3]^-$, 222.8 $[CuBr_2]^-$

Elemental analysis: Calculated (%) for $[(P_3C_2Mes_2)Br_5Cu_5K(CH_3CN)_3(dme)_{1.5}]$ (1370 g/mol): C 28.05, H 3.38, Br 29.16, N 3.07; found: C 27.52, H 3.20, Br 29.20, N 3.24.

Elemental analysis (after drying in vacuo for several hours) : Calculated (%) for $[(P_3C_2Mes_2)Br_5Cu_5K(CH_3CN)]$ (1153 g/mol): C 22.92, H 2.19, N 1.22; found: C 22.57, H 2.53, N 1.34.

1.4 Synthesis of $[(\mu,\eta^1:\eta^2:\eta^2\text{-}P_3C_2Mes_2)\{Cu(CH_3CN)(\mu_2\text{-}I)\}_4\{Cu(CH_3CN)_3\}]$

In a Schlenk tube a solution of $[K(P_3C_2Mes_2)]$ (50 mg, 0.12 mmol) in dme (9 mL) is first layered with a solvent mixture of dme and CH_3CN (1:1; 3 mL), afterwards with a solution of CuI (123 mg, 0.52 mmol) in CH_3CN (12 mL). After complete diffusion and precipitation of a beige powder the red solution is filtered and concentrated to 10 mL. While storing at -28°C the formation of small orange polyhedrons of $[(\mu,\eta^1:\eta^2:\eta^2\text{-}P_3C_2Mes_2)\{Cu(CH_3CN)(\mu_2\text{-}I)\}_4\{Cu(CH_3CN)_3\}]$ can be observed. The mother liquor is decanted, the crystals are washed with pentane (3 x 5 mL) and dried in vacuo. By concentrating the mother liquor to 2 mL and storing it at -28°C, a second crop of crystals can be obtained, which is isolated in the same way as before. $[(\mu,\eta^1:\eta^2:\eta^2\text{-}P_3C_2Mes_2)\{Cu(CH_3CN)(\mu_2\text{-}I)\}_4\{Cu(CH_3CN)_3\}]$ can also be synthesized by using thf instead of dme, however due to the lower solubility of $[K(P_3C_2Mes_2)]$ in thf a larger volume is needed (20 mL). $[(\mu,\eta^1:\eta^2:\eta^2\text{-}P_3C_2Mes_2)\{Cu(CH_3CN)(\mu_2\text{-}I)\}_4\{Cu(CH_3CN)_3\}]$ can also be synthesized by using $[Cp^*Fe(\eta^5\text{-}P_3C_2Mes_2)]$ as starting material.[3]

Analytical data of $[(\mu,\eta^1:\eta^2:\eta^2\text{-}P_3C_2Mes_2)\{Cu(CH_3CN)(\mu_2\text{-}I)\}_4\{Cu(CH_3CN)_3\}]$:

Yield: 55 mg (0.042 mmol, crystalline, 40% referred to CuI)

^{1}H NMR (CD_3CN): δ [ppm] = 1.95 (s, CH_3CN), 2.04 (s, 3H, *p*-CH_3), 2.13 (s, 3H, *p*-CH_3), 2.22 (s, 6H, *o*-CH_3), 2.27 (s, 6H, *o*-CH_3), 6.90 (s, 4H, *aryl*-H).

^{13}C{^{1}H} NMR (CD_3CN): δ [ppm] = 21.14, 23.50, 26.23, 128.66, 136.86, 138.37.

^{31}P{^{1}H} NMR (CD_3CN): δ [ppm] = 134 (br), 153.0 (br), 205 (br), 224 (br).

Positive ion ESI-MS (CH_3CN): *m/z* (%) = 2883.2 $[\{P_3C_2Mes_2\}_4Cu_{11}I_6]^+$, 2878.2 $[\{P_3C_2Mes_2\}_3Cu_{12}I_8]^+$, 2691.5 $[\{P_3C_2Mes_2\}_4Cu_{10}I_5]^+$, 2650.4 $[\{P_3C_2Mes_2\}_3Cu_{11}I_7]^+$, 2500.1 $[\{P_3C_2Mes_2\}_4Cu_9I_4]^+$, 2463.1 $[\{P_3C_2Mes_2\}_3Cu_{10}I_6]^+$, 2424.7 $[\{P_3C_2Mes_2\}_2Cu_{11}I_8]^+$, 2312.7 $[\{P_3C_2Mes_2\}_3Cu_{12}I_8]^+$, 2273.0 $[\{P_3C_2Mes_2\}_3Cu_9I_5]^+$, 2234.7 $[\{P_3C_2Mes_2\}_2Cu_{10}I_7]^+$, 2119.7 $[\{P_3C_2Mes_2\}_4Cu_7I_2]^+$, 2081.2 $[\{P_3C_2Mes_2\}_3Cu_8I_4]^+$, 2042.9 $[\{P_3C_2Mes_2\}_2Cu_9I_6]^+$, 1929.1 $[\{P_3C_2Mes_2\}_4Cu_6I]^+$, 1890.8 $[\{P_3C_2Mes_2\}_3Cu_7I_3]^+$, 1742.0 $[\{P_3C_2Mes_2\}_4Cu_5]^+$, 1700.9 $[\{P_3C_2Mes_2\}_3Cu_6I_2]^+$, 1661.8 $[\{P_3C_2Mes_2\}_2Cu_7I_4]^+$, 1511.0 $[\{P_3C_2Mes_2\}_3Cu_5I]^+$, 1472.4 $[\{P_3C_2Mes_2\}_2Cu_6I_3]^+$, 1321.6 $[\{P_3C_2Mes_2\}_3Cu_4]^+$, 1285.2 $[\{P_3C_2Mes_2\}_2Cu_5I_2]^+$, 1242.2 $[\{P_3C_2Mes_2\}Cu_6I_4]^+$, 1172.8 $[\{P_3C_2Mes_2\}_2Cu_4I\{CH_3CN\}_2]^+$, 1131.8 $[\{P_3C_2Mes_2\}_2Cu_4I\{CH_3CN\}]^+$, 1090.8 $[\{P_3C_2Mes_2\}_2Cu_4I]^+$, 1052.4 $[\{P_3C_2Mes_2\}Cu_5I_3]^+$

Negative ion ESI-MS (CH_3CN): *m/z* (%) = 1270.1 $[Cu_6I_7]^-$, 1078.2 $[Cu_5I_6]^-$, 888.3 $[Cu_4I_5]^-$, 698.5 $[Cu_3I_4]^-$, 506.6 $[Cu_2I_3]^-$, 316.8 (100) $[CuI_2]^-$

Elemental analysis: Calculated (%) for $[(P_3C_2Mes_2)Cu_5I_4(CH_3CN)_3]$ (1304 g/mol): C 23.96, H 2.40, N 3.22; found: C 23.96, H 2.50, N 3.28.

1.5 Synthesis of $[(\mu,\eta^1:\eta^3:\eta^3\text{-}P_3C_2Mes_2)_2\{Cu(CH_3CN)_3\}_2\{Cu(\mu_2\text{-}I)\}_6]$

In a Schlenk tube $[K(P_3C_2Mes_2)]$ (80 mg, 0.2 mmol) and CuI (190 mg, 1.0 mmol) are dissolved in a solvent mixture of dme (15 mL) and CH_3CN (3 mL). An immediate colour change to deep red and turbidity can be observed. The solution is stirred for 10 hours. After filtration the solvent of the red solution is removed in vacuo. The red solid is dissolved in a solvent mixture of CH_3CN (ca. 8 mL) and CH_2Cl_2 (ca. 8 mL), again filtered and layered with Et_2O. While storing at 8°C the formation of crystals of $[(\mu,\eta^1:\eta^3:\eta^3\text{-}P_3C_2Mes_2)_2\{Cu(CH_3CN)_3\}_2\{Cu(\mu_2\text{-}I)\}_6]$ can be observed. The mother liquor is decanted, the crystals are washed with hexane (3 x 5 mL) and dried in vacuo.
$[(\mu,\eta^1:\eta^3:\eta^3\text{-}P_3C_2Mes_2)_2\{Cu(CH_3CN)_3\}_2\{Cu(\mu_2\text{-}I)\}_6]$ can also be synthesized by using $[Cp^*Fe(\eta^5\text{-}P_3C_2Mes_2)]$ as starting material.3

Analytical data of $[(\mu,\eta^1:\eta^3:\eta^3\text{-}P_3C_2Mes_2)_2\{Cu(CH_3CN)_3\}_2\{Cu(\mu_2\text{-}I)\}_6]$:
Yield: 45 mg (0.021 mmol, crystalline, 17% referred to CuI)

^{1}H NMR (CD_3CN): δ [ppm] = 1.95 (s, CH_3CN), 2.04 (s, 3H, *p*-CH_3), 2.13 (s, 12H, *o*-CH_3), 2.20 (s, 6H, *o*-CH_3), 2.26 (s, 6H, *p*-CH_3), 6.90 (s, br, 6H, *aryl*-H).

^{31}P{^{1}H} NMR (CD_3CN): δ [ppm] = 135 (br), 206 (br), 223 (br).

Positive ion ESI-MS (CH_3CN): *m/z* (%) = 2693.1 $[\{P_3C_2Mes_2\}_4Cu_{10}I_5]^+$, 2501.1 $[\{P_3C_2Mes_2\}_4Cu_9I_4]^+$, 2462.7 $[\{P_3C_2Mes_2\}_3Cu_{10}I_6]^+$, 2311.2 $[\{P_3C_2Mes_2\}_4Cu_8I_3]^+$, 2274.7 $[\{P_3C_2Mes_2\}_3Cu_9I_5]^+$, 2119.2 $[\{P_3C_2Mes_2\}_4Cu_7I_2]^+$, 2082.8 $[\{P_3C_2Mes_2\}_3Cu_8I_4]^+$, 1929.4 $[\{P_3C_2Mes_2\}_4Cu_6I]^+$, 1891.0 $[\{P_3C_2Mes_2\}_3Cu_7I_3]^+$, 1740.1 $[\{P_3C_2Mes_2\}_4Cu_5]^+$, 1701.0 $[\{P_3C_2Mes_2\}_3Cu_6I_2]^+$, 1662.6 $[\{P_3C_2Mes_2\}_2Cu_7I_4]^+$, 1550.0 $[\{P_3C_2Mes_2\}_3Cu_5I\{CH_3CN\}]^+$, 1511.1 $[\{P_3C_2Mes_2\}_3Cu_5I]^+$, 1472.8 $[\{P_3C_2Mes_2\}_2Cu_6I_3]^+$, 1322.0 $[\{P_3C_2Mes_2\}_3Cu_4]^+$, 1283.6 $[\{P_3C_2Mes_2\}_2Cu_5I_2]^+$, 1173.0 $[\{P_3C_2Mes_2\}_2Cu_4I\{CH_3CN\}_2]^+$, 1131.9 $[\{P_3C_2Mes_2\}_2Cu_4I\{CH_3CN\}]^+$, 1093.0 $[\{P_3C_2Mes_2\}_2Cu_4I]^+$, 996.2 $[\{P_3C_2Mes_2\}_4Cu_7I]^{2+}$, 901.2 $[\{P_3C_2Mes_2\}_4Cu_6]^{2+}$

Negative ion ESI-MS (CH_3CN): *m/z* (%) = 2562.5 $[\{P_3C_2Mes_2\}_4Cu_8I_5]^-$, 2528.7 $[\{P_3C_2Mes_2\}_3Cu_9I_7]^-$, 2490.2 $[\{P_3C_2Mes_2\}_2Cu_{10}I_9]^-$, 2373.2 $[\{P_3C_2Mes_2\}_4Cu_7I_4]^-$, 2334.9 $[\{P_3C_2Mes_2\}_3Cu_8I_6]^-$, 2296.4 $[\{P_3C_2Mes_2\}_2Cu_9I_8]^-$, 2144.7 $[\{P_3C_2Mes_2\}_3Cu_7I_5]^-$, 2106.7 $[\{P_3C_2Mes_2\}_2Cu_8I_7]^-$, 2070.2 $[\{P_3C_2Mes_2\}Cu_9I_9]^-$, 1954.9 $[\{P_3C_2Mes_2\}_3Cu_6I_4]^-$, 1916.5

$[\{P_3C_2Mes_2\}_2Cu_7I_6]^-$, 1878.3 $[\{P_3C_2Mes_2\}Cu_8I_8]^-$, 1841.8 $[Cu_9I_{10}]^-$, 1765.0 $[\{P_3C_2Mes_2\}_3Cu_5I_3]^-$, 1726.7 $[\{P_3C_2Mes_2\}_2Cu_6I_5]^-$, 1688.3 $[\{P_3C_2Mes_2\}Cu_7I_7]^-$, 1651.8 $[Cu_8I_9]^-$, 1534.7 $[\{P_3C_2Mes_2\}_2Cu_5I_4]^-$, 1498.3 $[\{P_3C_2Mes_2\}Cu_6I_6]^-$, 1460.1 $[Cu_7I_8]^-$, 1306.5 $[\{P_3C_2Mes_2\}Cu_5I_5]^-$, 1270.1 $[Cu_6I_7]^-$, 1116.6 $[\{P_3C_2Mes_2\}Cu_4I_4]^-$, 1078.2 $[Cu_5I_6]^-$, 888.5 $[Cu_4I_5]^-$, 698.5 $[Cu_3I_4]^-$, 506.6 $[Cu_2I_3]^-$, 316.6 (100) $[CuI_2]^-$

Elemental analysis: Calculated (%) for $[(P_3C_2Mes_2)_2Cu_8I_6(CH_3CN)_4]$ (2145 g/mol): C 26.88, H 2.63, N 2.61; found: C 26.59, H 2.69, N 2.70.

1.6 Synthesis of 5

In a Schlenk tube a solution of $[K(P_3C_2Mes_2)]$ (16 mg, 0.042 mmol) in dme (5 mL) is layered with a solution of CuI (25 mg, 0.13 mmol) in CH_3CN (5 mL). After complete diffusion the red solution is concentrated to 5 mL and layered with hexane. The formation of small red-brown blocks of **5** at the phase boundary could be observed once. The mother liquor was decanted, the crystals were washed with hexane (3 x 5 mL) and dried in vacuo. Attempts to reproduce **5** failed every time.

Analytical data of **5**:

Yield: a few crystals (< 5%)

Positive ion ESI-MS (CH_3CN): *m/z* (%) = 2692.5 $[\{P_3C_2Mes_2\}_4Cu_{10}I_5]^+$, 2500.6 $[\{P_3C_2Mes_2\}_4Cu_9I_4]^+$, 2309.5 $[\{P_3C_2Mes_2\}_4Cu_8I_3]^+$, 2119.5 $[\{P_3C_2Mes_2\}_4Cu_7I_2]^+$, 2079.1 $[\{P_3C_2Mes_2\}_3Cu_8I_4]^+$, 1929.4 $[\{P_3C_2Mes_2\}_4Cu_6I]^+$, 1891.0 $[\{P_3C_2Mes_2\}_3Cu_7I_3]^+$, 1739.6 $[\{P_3C_2Mes_2\}_4Cu_5]^+$, 1700.9 $[\{P_3C_2Mes_2\}_3Cu_6I_2]^+$, 1552.1 $[\{P_3C_2Mes_2\}_3Cu_5I\{CH_3CN\}]^+$, 1511.1 $[\{P_3C_2Mes_2\}_3Cu_5I]^+$, 1472.6 $[\{P_3C_2Mes_2\}_2Cu_6I_3]^+$, 1319.2 (100) $[\{P_3C_2Mes_2\}_3Cu_4]^+$, 1172.8 $[\{P_3C_2Mes_2\}_2Cu_4I\{CH_3CN\}_2]^+$, 1131.9 $[\{P_3C_2Mes_2\}_2Cu_4I\{CH_3CN\}]^+$, 1090.9 $[\{P_3C_2Mes_2\}_2Cu_4I]^+$, 901.0 $[\{P_3C_2Mes_2\}_4Cu_6]^{2+}$

Negative ion ESI-MS (CH_3CN): *m/z* (%) = 888.4 $[Cu_4I_5]^-$, 698.5 $[Cu_3I_4]^-$, 506.6 $[Cu_2I_3]^-$, 316.8 (100) $[CuI_2]^-$

1.7 Synthesis of 6

A solution of $[K(P_3C_2Mes_2)]$ (33 mg, 0.084 mmol) in dme (15 mL) is added to a solution of CuI (160 mg, 0.84 mmol) in CH_3CN (15 mL), whereas the solution turned deep red. It was stirred for three hours, filtered and layered with Et_2O. After several months at r.t. orange crystals of **6** at the upper level have formed. The mother liquor was decanted, the crystals were washed with hexane (3 x 5 mL) and dried in vacuo.

Analytical data of **6**:

Yield: 86 mg (0.05 mmol, crystalline, 60%)

^{1}H NMR (CD_3CN): δ [ppm] = 1.11 (t, Et_2O), 1.95 (s, CH_3CN), 2.26 (s, 6H, *o*-CH_3), 2.28 (s, 3H, *p*-CH_3), 3.28 (s, dme), 3.41 (q, Et_2O), 3.44 (s, dme), 6.93 (s, 2H, *aryl*-H).

^{31}P{^{1}H} NMR (CD_3CN): δ [ppm] = 136 (br, 2P), 222 (br, 1P).

Negative ion ESI-MS (dme, CH_3CN, Et_2O): *m/z* (%) = 888.3 $[Cu_4I_5]^-$, 698.4 $[Cu_3I_4]^-$, 506.6 $[Cu_2I_3]^-$, 316.7 (100) $[CuI_2]^-$

Elemental analysis: Calculated (%) for $[(P_3C_2Mes_2)I_7Cu_6(CH_3CN)_4]$ (1816 g/mol): C 19.84, H 2.05, N 3.08; found: C 20.92, H 2.20, N 3.16.

1.8 Synthesis of 7

A solution of $[K(P_3C_2{}^tBu_2)]$ (50 mg, 0.18 mmol) in dme (10 mL) is added to a solution of CuCl (55 mg, 0.56 mmol) in CH_3CN (10 mL). Within one minute the colour changes from yellow to orange up to red and becomes turbid after 30 minutes of stirring. After two hours the reaction mixture is filtered, the solvent removed in vacuo and the red solid dissolved in as less as possible CH_3CN. By cooling the formation of neon

orange plates of **7** is observed. The mother liquor is decanted, the crystals are washed with hexane (3 x 5 mL) and dried in vacuo.

Analytical data of **7**:

Yield: 65 mg (0.044 mmol, 73% referred to [K($P_3C_2{}^tBu_2$)])

^{1}H NMR (CD_3CN): δ [ppm] = 1.77 (s, ^{t}Bu), 1.99 (s, CH_3CN).

^{31}P{^{1}H} NMR (CD_3CN): δ [ppm] = 136.7 (s, br, 2P), 264.8 (s, br, 1P).

Positive ion ESI-MS (CH_3CN): *m/z* (%) = 2918.8 [$\{P_3C_2{}^tBu_2\}_7Cu_{16}Cl_8$]$^+$, 2821.0 [$\{P_3C_2{}^tBu_2\}_7Cu_{15}Cl_7$]$^+$, 2725.0 [$\{P_3C_2{}^tBu_2\}_7Cu_{14}Cl_6$]$^+$, 2621.2 [$\{P_3C_2{}^tBu_2\}_6Cu_{15}Cl_8$]$^+$, 2523.0 [$\{P_3C_2{}^tBu_2\}_6Cu_{14}Cl_7$]$^+$, 2425.0 [$\{P_3C_2{}^tBu_2\}_5Cu_{15}Cl_9$]$^+$, 2327.0 [$\{P_3C_2{}^tBu_2\}_5Cu_{14}Cl_8$]$^+$, 2227.1 [$\{P_3C_2{}^tBu_2\}_5Cu_{13}Cl_7$]$^+$, 2129.0 [$\{P_3C_2{}^tBu_2\}_5Cu_{12}Cl_6$]$^+$, 2030.9 [$\{P_3C_2{}^tBu_2\}_5Cu_{11}Cl_5$]$^+$, 1935.0 [$\{P_3C_2{}^tBu_2\}_4Cu_{12}Cl_7$]$^+$, 1835.0 [$\{P_3C_2{}^tBu_2\}_4Cu_{11}Cl_6$]$^+$, 1185.9 [$\{P_3C_2{}^tBu_2\}_3Cu_6Cl_2\{CH_3CN\}$]$^+$, 1144.9 [$\{P_3C_2{}^tBu_2\}_3Cu_6Cl_2$]$^+$, 1046.9 [$\{P_3C_2{}^tBu_2\}_3Cu_5Cl$]$^+$, 947.1 (100) [$\{P_3C_2{}^tBu_2\}_3Cu_4$]$^+$,

Negative ion ESI-MS (CH_3CN): *m/z* (%) = 2988.3 [$\{P_3C_2{}^tBu_2\}_7Cu_{18}Cl_{13}$]$^+$, 2891.1 [$\{P_3C_2{}^tBu_2\}_7Cu_{17}Cl_{12}$]$^+$, 2792.8 [$\{P_3C_2{}^tBu_2\}_6Cu_{16}Cl_{11}$]$^+$, 2694.8 [$\{P_3C_2{}^tBu_2\}_6Cu_{15}Cl_{10}$]$^+$, 2595.0 [$\{P_3C_2{}^tBu_2\}_6Cu_{14}Cl_9$]$^+$, 2498.9 [$\{P_3C_2{}^tBu_2\}_5Cu_{15}Cl_{11}$]$^+$, 2398.9 [$\{P_3C_2{}^tBu_2\}_5Cu_{14}Cl_{10}$]$^+$, 2298.9 [$\{P_3C_2{}^tBu_2\}_5Cu_{13}Cl_9$]$^+$, 2201.0 [$\{P_3C_2{}^tBu_2\}_4Cu_{14}Cl_{11}$]$^+$, 2102.9 [$\{P_3C_2{}^tBu_2\}_4Cu_{13}Cl_{10}$]$^+$, 2004.9 [$\{P_3C_2{}^tBu_2\}_3Cu_{14}Cl_{12}$]$^+$, 1906.8 [$\{P_3C_2{}^tBu_2\}_3Cu_{13}Cl_{11}$]$^+$, 1806.8 [$\{P_3C_2{}^tBu_2\}_3Cu_{12}Cl_{10}$]$^+$, 1708.9 [$\{P_3C_2{}^tBu_2\}_3Cu_{11}Cl_9$]$^+$, 1610.9 [$\{P_3C_2{}^tBu_2\}_3Cu_{10}Cl_8$]$^+$, 1512.9 [$\{P_3C_2{}^tBu_2\}_2Cu_{11}Cl_{10}$]$^+$, 1412.7 [$\{P_3C_2{}^tBu_2\}_2Cu_{10}Cl_9$]$^+$, 1314.8 [$\{P_3C_2{}^tBu_2\}_2Cu_9Cl_8$]$^+$, 1216.6 [$\{P_3C_2{}^tBu_2\}_2Cu_8Cl_7$]$^+$, 1118.6 [$\{P_3C_2{}^tBu_2\}_2Cu_7Cl_6$]$^+$, 1020.7 [$\{P_3C_2{}^tBu_2\}Cu_8Cl_8$]$^+$, 920.7 [$\{P_3C_2{}^tBu_2\}Cu_7Cl_7$]$^+$, 822.6 [$\{P_3C_2{}^tBu_2\}Cu_6Cl_6$]$^+$, 724.6 [$\{P_3C_2{}^tBu_2\}Cu_5Cl_5$]$^+$, 624.8 [$\{P_3C_2{}^tBu_2\}Cu_4Cl_4$]$^+$, 528.7 [$\{P_3C_2{}^tBu_2\}Cu_3Cl_3$]$^+$, 428.9 [$\{P_3C_2{}^tBu_2\}Cu_2Cl_2$]$^+$, 328.9 [$\{P_3C_2{}^tBu_2\}CuCl$]$^+$, 232.7 [$Cu_2Cl_3$]$^-$

Elemental analysis: Calculated (%) for [$(P_3C_2{}^tBu_2)_3Cl_6Cu_9(CH_3CN)_2$] (1560 g/mol): C 26.17, H 3.88, N 1.80; found: C 26.02, H 3.74, N 1.73.

1.9 Synthesis of 8

In a Schlenk tube a solution of [K($P_3C_2{}^tBu_2$)] (30 mg, 0.11 mmol) in dme (15 mL) is layered with a solution of CuCl (55 mg, 0.55 mmol) in CH_3CN (10 mL). Already after several hours the formation of big dark red blocks of **8** at the red phase boundary is observed. After complete diffusion the mother liquor is decanted, the crystals are washed with hexane (3 x 5 mL) and dried in vacuo.

Analytical data of **8**:

Yield: 56 mg (0.031 mmol, 85% referred to [K($P_3C_2{}^tBu_2$)])

Negative ion ESI-MS (mother liquor, dme/CH_3CN): *m/z* (%) = 232.6 [Cu_2Cl_3]$^-$, 134.7 [$CuCl_2$]$^-$

Elemental analysis: Calculated (%) for [$(P_3C_2{}^tBu_2)_3Cl_7Cu_{10}(CH_3CN)_3(C_4H_{10}O_2)$] (1790 g/mol): C 26.83, H 4.11, Cl 13.86, N 2.35; found: C 26.61, H 4.00, Cl 13.84, N 2.51.

1.10 Synthesis of 9

In a Schlenk tube a solution of [K($P_3C_2{}^tBu_2$)] (30 mg, 0.11 mmol) in dme (15 mL) is layered with a solution of CuBr (80 mg, 0.55 mmol) in CH_3CN (10 mL). Already after several hours the formation of big dark red blocks of **9** at the red phase boundary is observed. After complete diffusion the mother liquor is decanted, the crystals are washed with hexane (3 x 5 mL) and dried in vacuo.

Analytical data of **9**:

Yield: 50 mg (0.024 mmol, 65% referred to [K($P_3C_2{}^tBu_2$)])

Positive ion ESI-MS (thf/CH_3CN): *m/z* (%) = 3415.1 $[\{P_3C_2{}^tBu_2\}_7Cu_{17}Br_9]^+$, 3267.9 $[\{P_3C_2{}^tBu_2\}_7Cu_{16}Br_8]^+$, 3123.8 $[\{P_3C_2{}^tBu_2\}_6Cu_{16}Br_9]^+$, 2979.9 $[\{P_3C_2{}^tBu_2\}_6Cu_{15}Br_8]^+$, 2834.7 $[\{P_3C_2{}^tBu_2\}_6Cu_{14}Br_7]^+$, 2688.8 $[\{P_3C_2{}^tBu_2\}_6Cu_{13}Br_6]^+$, 2541.2 $[\{P_3C_2{}^tBu_2\}_5Cu_{13}Br_7]^+$, 2399.3 $[\{P_3C_2{}^tBu_2\}_5Cu_{12}Br_6]^+$, 2253.1 $[\{P_3C_2{}^tBu_2\}_5Cu_{11}Br_5]^+$, 1672.9 $[\{P_3C_2{}^tBu_2\}_4Cu_8Br_3]^+$, 1526.5 $[\{P_3C_2{}^tBu_2\}_4Cu_7Br_2]^+$, 1385.1 $[\{P_3C_2{}^tBu_2\}_4Cu_6Br]^+$, 1234.8 $[\{P_3C_2{}^tBu_2\}_3Cu_6Br_2]^+$, 1090.9 $[\{P_3C_2{}^tBu_2\}_3Cu_5Br]^+$, 947.0 $[\{P_3C_2{}^tBu_2\}_3Cu_4]^+$

Negative ion ESI-MS (thf/CH_3CN): *m/z* (%) = 3419.3 $[\{P_3C_2{}^tBu_2\}_5Cu_{18}Br_{14}]^-$, 3281.9 $[\{P_3C_2{}^tBu_2\}_6Cu_{16}Br_{11}]^-$, 3136.0 $[\{P_3C_2{}^tBu_2\}_6Cu_{15}Br_{10}]^-$, 2993.8 $[\{P_3C_2{}^tBu_2\}_6Cu_{14}Br_9]^-$, 2843.2 $[\{P_3C_2{}^tBu_2\}_5Cu_{14}Br_{10}]^-$, 2701.4 $[\{P_3C_2{}^tBu_2\}_5Cu_{13}Br_9]^-$, 2557.1 $[\{P_3C_2{}^tBu_2\}_4Cu_{13}Br_{10}]^-$, 2409.0 $[\{P_3C_2{}^tBu_2\}_4Cu_{12}Br_9]^-$, 2262.9 $[\{P_3C_2{}^tBu_2\}_4Cu_8Br_{11}]^-$, 2114.8 $[\{P_3C_2{}^tBu_2\}_3Cu_{11}Br_9]^-$, 1968.5 $[\{P_3C_2{}^tBu_2\}_3Cu_{10}Br_8]^-$, 1826.3 $[\{P_3C_2{}^tBu_2\}_3Cu_9Br_7]^-$, 1680.5 $[\{P_3C_2{}^tBu_2\}_3Cu_8Br_6]^-$, 1538.6 $[\{P_3C_2{}^tBu_2\}_3Cu_7Br_5]^-$, 1386.3 $[\{P_3C_2{}^tBu_2\}_2Cu_7Br_6]^-$, 1242.4 $[\{P_3C_2{}^tBu_2\}_2Cu_6Br_5]^-$, 1092.3 $[\{P_3C_2{}^tBu_2\}Cu_6Br_6]^-$, 519.0 $[\{P_3C_2{}^tBu_2\}Cu_2Br_2]^-$, 366.7 $[Cu_2Br_3]^-$, 222.9 $[CuBr_2]^-$

Negative ion ESI-MS (mother liquor, dme/CH_3CN): *m/z* (%) = 366.4 $[Cu_2Br_3]^-$, 222.6 $[CuBr_2]^-$

Elemental analysis: Calculated (%) for $[(P_3C_2{}^tBu_2)_3Br_7Cu_{10}(CH_3CN)_3]$ (2011 g/mol): C 21.50, H 3.16, Br 27.81, N 2.09; found: C 21.89, H 3.45, Br 26.73, N 1.65.

Elemental analysis (after two years in air): Calculated (%) for $[(P_3C_2{}^tBu_2)_3Br_7Cu_{10}(CH_3CN)]$ (1929 g/mol): C 19.92, H 2.98, N 0.73; found: C 19.83, H 3.48, N 0.92.

1.11 Synthesis of 10

In a Schlenk tube $[K(P_3C_2{}^tBu_2)]$ (50 mg, 0.18 mmol) and CuI (105 mg, 0.55 mmol) are dissolved in thf (12 mL). An immediate colour change to deep red can be observed. The solution is stirred for 2 hours, before the solvent is removed in vacuo. The red solid is dissolved in as less CH_3CN as possible and layered onto toluene. After complete diffusion the mother liquor is almost colourless and dark red blocks of **10** have formed. The mother liquor is decanted, the crystals are washed with toluene (3 x 5 mL) and dried in vacuo. Attempts to reproduce **10** were successful only once.

Analytical data of **10**:

Yield: 13 mg (0.006 mmol, 8% referred to CuI)

Negative ion ESI-MS (CH_3CN): *m/z* (%) = 1078.1 $[Cu_5I_6]^-$, 888.3 $[Cu_4I_5]^-$, 698.5 $[Cu_3I_4]^-$, 506.6 $[Cu_2I_3]^-$, 316.7 (100) $[CuI_2]^-$

2. X-ray Structure Analysis

The compounds including co-crystallized solvent molecules characterized with X-ray structure analysis are

**2**	$[(P_3C_2Mes_2)_2\{Cu(CH_3CN)\}_2\{Cu(CH_3CN)_2\}]Cl\cdot0.6MeCN$
**3**	$K(dme))[Cu_4Cl_4(MeCN)_4(P_3C_2Mes_2)_3(CuCl)(MeCN)_{0.5}]\cdot1.5MeCN\cdot0.75dme$
**4**	$K(thf)[(P_3C_2Mes_2)(Cu(MeCN)Br)_4(CuBr)]\cdot1.3MeCN$
**5**	$[Cu_5I_4(MeCN)_4(P_3C_2Mes_2)]$
**6**	$[Cu_6I_7(MeCN)_5(P_3C_2Mes_2)]\cdot0.5MeCN\cdot0.5C_6H_{14}\cdot0.4Et_2O$
**7**	$[Cu_6Cl_5(MeCN)_3(P_3C_2(tBu)_2)_3(CuCl)(Cu(MeCN)_3)_2]\cdot8.1MeCN$
**8**	$[Cu_6Cl_4(P_3C_2(tBu)_2)_3(CuCl_{0.4}(MeCN)_{0.6})(Cu(MeCN)_3)_{0.25}(Cu_{0.867}Cl_{0.817}(MeCN)_{0.183})_3]$
**9**	$[Cu_6Br_4(P_3C_2(tBu)_2)_3(CuBr_{0.5}(MeCN)_{0.5})(Cu(MeCN)_3)_{0.1}(CuBr_{0.867}(MeCN)_{0.133})_3]\cdot0.367MeCN$
**10**	$(K(thf)_6)[Cu_7I_6(MeCN)_4(P_3C_2(tBu)_2)_2]\cdot0.5thf$

Crystals of **2-10** were taken from a Schlenk tube under a stream of argon and covered with mineral oil. The single crystal was taken to the pre-centered goniometer head with CryoMount® and directly attached to the diffractometer into a stream of cold nitrogen. The data for **6** were collected on an Agilent Technologies diffractometer equipped with Titan^{S2} CCD detector and a SuperNova CuK_{α} microfocus source using 1° ω scans. The data for **2**, **3, 4**, **5, 9** were collected using 1° ω scans and for **7, 8** and **10** using 0.5° ω scans on an Agilent Technologies diffractometer equipped with Atlas CCD detector and a SuperNova CuK_{α} microfocus source. All measurements were performed at 123 K. Crystallographic data and details of the diffraction experiments are given in Table S 1-Table S 3. The structures of **2-8, 10** were solved by direct methods with *SIR97*,[4] *SHELX97* or *SHELX2013*.[5] The structures were refined by full-matrix least-squares method against $|F|^2$ in anisotropic approximation using either *SHELXL97* or the multiprocessor and variable memory version *SHELXL2013*. All non-hydrogen atoms were refined anisotropically, while the hydrogen atoms were refined riding on pivot atoms.

2: The structure was refined as an inversion twin with batch components 0.8(1)/0.2(1). We faced with unexplainably large displacement parameters for almost all atoms even at T=123K and quite bad quality factors in **2** despite the signs for a good experiment, clear symmetry (for detailed information: see Section 3) and a chemically reasonable structure of the complex. Despite this, only positions of two adjacent P atoms of the phospholyl ligands were split with the probability 0.7/0.3. The outer sphere counter anion Cl^- is disordered over six close positions and therefore was refined in an isotropic approximation. The solvent molecules and one of the coordinated MeCN molecules are disordered over 2 and 3 positions, respectively. They were refined with geometric restraints.

3: The CuCl and Cu(MeCN)Cl fragments of the Cu_2Cl_2 bridges in **3** are disordered (1:1) over several close positions, probably because of a mixed trigonally and tetrahedrally coordinated Cu. The coordinated to K^+ and solvated *dme* molecules as well as the solvated MeCN molecules are disordered.

4: In **4** the Cu_2Br_2 fragment is disordered around the direction of the chain so that there are three close positions of the Cu atom in the independent part. The corresponding Br atoms are disordered over total eight positions. The Cu and Br atoms have occupancies less than 0.2 and were therefore refined in an isotropic approximation. The residual electron density peaks of more than 2 $e \cdot A^{-3}$ can only be avoided if the disorder of the Cu_2Br_2 fragment is described with such a high number of the split positions. The main factors that favor the disorder are presumably 1) a high steric demand of two Mes groups, which screen the disordered fragment and allow it to re-orientate freely in the resulted cavity, and 2) the choice between a trigonal and tetrahedral coordination environment of Cu that in some of disordered positions can additionally coordinate solvated MeCN molecule. The solvent MeCN molecules and thf molecules coordinated to K^+ counter cation are disordered over 2 positions and were treated with some geometrical restraints.

5: The diffraction pattern of crystal **5** was featured by diffuse scattering (see Section 4) that is described below. The effect is strongly related to the disorder of the repeating units over 4-axis.

6: In **6** one of the MeCN molecules coordinated to copper is disordered over two close positions. Solvated MeCN and Et_2O were refined as 0.5 and 0.4 molecular occupancies, and their positions partly overlap. The hexane molecule is disordered over the inversion center.

7: In **7** the Cl atoms of the bridging $Cu_6Cl_5(P_3C_2(t\text{Bu})_2)_3$ fragments are disordered over 3 close positions. One MeCN molecule coordinated to copper is disordered over two positions. Solvated MeCN is severely disordered in the inter-chain cavities.

8 and **9**: Compound **8** and **9** are isotypical, and the model of **9** was used for the refinement of **8**. The similar structures however differ in details. In the Cl analogue the copper atom and the Cl atom bonded to it are disordered over two positions (Fig. S), while in the Br analogue it is ordered (Fig. S). This difference leads to a variation of the polymeric chain structure. Another Cu atom belonging to the $Cu(MeCN)_3$ fragment occupies the position with different probability refined to 0.25 for the Cl and to 0.1 for the Br analogues, respectively. In both structures the Cu atoms in the Cu(2) position are bonded with X(2) (X=Cl, Br) that has less occupancy than Cu(2). We supposed that the environment of Cu(2) is most probably completed by a coordinated MeCN molecule (minor content of 0.183 or 0.133, respectively) that was not located in the mixed X/MeCN positions because of the proximity to the heavy X = Cl or Br atoms. The undetermined MeCN was nevertheless included into the final structural formula.

According to the Cambridge Structural Database, at least four crystal structures are known, where a MeCN ligand is coordinated in a monodentate-bridging mode to two Cu atoms. Three of them are complexes of Cu^{+} with N- and P-donor atom ligands and one is the complex of Cu^{2+}. Two Cu-μ-N distances in $[Cu(dpme)_2(\mu\text{-}NCMe)(NCMe)_2]ClO_4\cdot MeCN$ of 2.29 and 2.32 Å, are close to the Cu-X(2) distances in **8** and **9**.[6] The atomic coordinates of the $\{Cu_2(\mu\text{-}NCMe)\}$ fragment were used to model a possible MeCN bridging ligand with coinciding positions of X(2) and N atoms. The analysis of the interatomic distances involving the atoms of the modelled MeCN proves that the corresponding cavity in both crystal structures is large enough to allow this ligand to occupy the mixed ligand X/MeCN position.

10: In **10** four of six thf molecules in the $K(thf)_6^{+}$ cation are conformationally disordered over two positions. The solvated thf molecule is disordered in the space between polymeric layers.

CIF files with comprehensive information on the details of the diffraction experiments and full tables of bond lengths and angles for the products are deposited in Cambridge Crystallographic Data Centre under the deposition codes CCDC-1056476- CCDC-1056484, respectively. The topological features of the 3D polymers were performed using TOPOSPro[7] (see Section 5).

Table S 1. Experimental details for compounds **2-4**

	**2**	**3**	**4**
CCDC Codes	CCDC-1056476	CCDC-1056477	CCDC-1056478
Chemical formula	$C_{48}H_{56}ClCu_3N_4P_6\cdot 0.6(CH_3CN)$	$C_{39}H_{57.50}Cl_5Cu_5KN_6O_{3.50}P_3$	$C_{34.60}H_{45.90}Br_5Cu_5KN_{5.30}OP_3$
M_r	1125.49	1293.37	1401.32
Crystal system, space group	Tetragonal, $I\bar{4}2d$	Monoclinic, $P2_1/n$	Monoclinic, $C2/m$
Temperature (K)	123	150	123
a, *b*, *c* (Å)	25.9670 (2), 17.9760 (2)	13.5753 (3), 26.0211 (6), 15.6295 (4)	19.5159(3), 25.8364(4), 13.6766(2)
α, β, γ (°)	12120.9 (2)	97.061 (2)	90, 127.405(1), 90
V (Å^3)	8	5479.2 (2)	5477.93(16)
Z	4634	4	4
F(000)	1.234	2622	2730
Radiation type	Cu *K*α	Cu *K*α	Cu *K*α
μ (mm^{-1})	3.38	6.22	8.16
Crystal colour and shape	red prism	yellow prism	yellow-orange block

Crystal size (mm)	0.18 × 0.13 × 0.03	0.11 × 0.05 × 0.04	0.09 × 0.06 × 0.04
Data collection			
Diffractometer	SuperNova, Single source at offset, Atlas diffractometer	SuperNova, Single source at offset, Atlas diffractometer	SuperNova, Single source at offset, Atlas diffractometer
Absorption correction	Gaussian	Gaussian	Analytical
T_{min}, T_{max}	0.648, 0.893	0.601, 0.778	0.569, 0.761
No. of measured, independent and observed [$I > 2\sigma(I)$] reflections	13152, 5757, 4063	20040, 10599, 6315	34635, 5576, 4748
R_{int}	0.022	0.041	0.030
$(\sin \theta/\lambda)_{max}$ (Å^{-1})	0.622	0.622	0.623
Refinement			
$R[F^2 > 2\sigma(F^2)]$, $wR(F^2)$, S	0.095, 0.260, 1.05	0.044, 0.108, 0.88	0.043, 0.139, 1.11
No. of reflections	5757	10599	5576
No. of parameters	353	608	312
No. of restraints	12	-	-
H-atom treatment	H-atom parameters constrained	H-atom parameters constrained	H-atom parameters constrained
$\Delta\rangle_{max}$, $\Delta\rangle_{min}$ (e Å^{-3})	1.86, -0.54	0.57, -0.43	1.80, -0.79

Computer programs: *CrysAlis PRO*, Agilent Technologies, *SIR97* (Altomare, 1999), *SHELXL97* (Sheldrick, 1997), *PLATON* (Spek, 1990), *PLATON* (Spek, 2003).

Table S 2. Experimental details for compounds **5-7**

	**5**	**6**	**7**
CCDC Codes	CCDC-1056479	CCDC-1056480	CCDC-1056481
Chemical formula	$C_{28}H_{34}Cu_5I_4N_4P_3$	$C_{35.6}H_{49.5}Cu_7I_6N_{5.5}O_{0.4}P_3$	$C_{64.20}H_{105.30}Cl_6Cu_9N_{17.10}P_9$
M_r	1344.80	1860.00	2180.05
Crystal system, space group	Tetragonal, $P4/n$	Triclinic, $P\bar{1}$	Monoclinic, $P2_1/m$
Temperature (K)	123	123	123
a, b, c (Å)	15.5501(2), 9.3497(2)	11.9259(10), 12.3005(7), 20.8887(11)	13.1219 (2), 27.5544 (6), 13.9810 (4)
α, β, γ (°)	90, 90, 90	79.096(5), 89.381(5), 64.029(7)	95.626 (2)
V (Å^3)	2260.81(8)	2696.1(3)	5030.7 (2)
Z	2	2	2
F(000)	1264	1742	2216
Radiation type	Cu $K\alpha$	Cu $K\alpha$	Cu $K\alpha$
μ (mm^{-1})	25.21	31.04	5.18
Crystal colour and shape	red-brown block	orange lath	orange plate
Crystal size (mm)	0.08 × 0.06 × 0.03	0.24 × 0.09 × 0.05	0.12 × 0.09 × 0.04
Data collection			
Diffractometer	SuperNova, Single source at offset, Atlas diffractometer	SuperNova, TitanS2 diffractometer	SuperNova, Single source at offset, Atlas diffractometer
Absorption correction	Gaussian	Gaussian	Analytical
T_{min}, T_{max}	0.354, 0.540	0.093, 0.550	0.693, 0.857
No. of measured, independent and observed [$I > 2\sigma(I)$] reflections	5373, 2050, 1615	16228, 10356, 8315	17849, 9906, 7571
R_{int}	0.035	0.049	0.032
$(\sin \theta/\lambda)_{max}$ (Å^{-1})	0.605	0.624	0.623
Refinement			
$R[F^2 > 2\sigma(F^2)]$, $wR(F^2)$, S	0.076, 0.220, 1.06	0.051, 0.142, 1.01	0.047, 0.143, 1.03
No. of reflections	2050	10356	9906
No. of parameters	193	534	569
No. of restraints	0	12	0
H-atom treatment	H-atom parameters not refined	H-atom parameters constrained	H-atom parameters constrained
$\Delta\rangle_{max}$, $\Delta\rangle_{min}$ (e Å^{-3})	1.69, -1.24	2.46, -2.62	0.96, -0.62

Computer programs: *CrysAlis PRO*, Agilent Technologies, *SIR97* (Altomare, 1999), *SHELXL97* (Sheldrick, 1997), *PLATON* (Spek, 1990), *PLATON* (Spek, 2003).

Table S 3. Experimental details for compounds **8-10**

	**8**	**9**	**10**
CCDC Codes	CCDC-1056482	CCDC-1056483	CCDC-1056484
Chemical formula	$C_{33.8}H_{59.67}Cl_{6.85}Cu_{9.85}N_{1.9}P_9$	$C_{33.13}H_{58.7}Br_{7.10}Cu_{10.10}P_9N_{1.57}$	$C_{46}H_{88}Cu_7I_6KO_{6.50}P_6$
M_r	1640.10	1966.89	2176.26
Crystal system, space group	Cubic, $F\bar{4}3c$	Cubic, $F\bar{4}3c$	Orthorhombic, *Pbcn*
Temperature (K)	123	123	123
a, *b*, *c* (Å)	35.5021(1)	35.7790(1)	13.3881(3), 29.8918(6), 18.0577(3)
V (Å^3)	44746.8(4)	45802.0(4)	7226.6(2)
Z	32	32	4
F(000)	26011	30236	4184
Radiation type	Cu $K\alpha$	Cu $K\alpha$	Cu $K\alpha$
μ (mm^{-1})	9.651	12.383	24.429
Crystal colour and shape	dark red block	dark red block	dark brown block
Crystal size (mm)	0.19 × 0.14 × 0.13	0.18 × 0.16 × 0.10	0.24 × 0.20 × 0.12
Data collection			
Diffractometer	SuperNova, Single source at offset, Atlas diffractometer	SuperNova, Single source at offset, Atlas diffractometer	SuperNova, Single source at offset, Atlas diffractometer
Absorption correction	Gaussian	Analytical	Gaussian
T_{min}, T_{max}	0.325, 0.508	0.211, 0.427	0.033, 0.211
No. of measured, independent and observed [$I > 2\sigma(I)$] reflections	48284, 3910, 3826	91270, 3957, 3934	34111, 7067, 6829
R_{int}	0.031	0.024	0.095
$(\sin\theta/\lambda)_{max}$ (Å^{-1})	0.630	0.627	0.622
Refinement			
$R[F^2 > 2\sigma(F^2)]$, $wR(F^2)$, S	0.046, 0.132, 1.07	0.030, 0.0875, 1.069	0.080, 0.2173, 1.097
No. of reflections	3910	3957	7067
No. of parameters	207	197	338
H-atom treatment	H-atom parameters constrained	H-atom parameters constrained	H-atom parameters constrained
$\Delta\rangle_{max}$, $\Delta\rangle_{min}$ (e Å^{-3})	2.14, -0.95	1.50, -1.25	2.14, -1.13
Absolute structure parameter	0.020 (13)	-0.027(5)	-

Computer programs: *CrysAlis PRO*, Agilent Technologies, *SIR97* (Altomare, 1999), *SHELXL97* (Sheldrick, 1997), *SHELXL2014* (Sheldrick, 2014), *PLATON* (Spek, 2003).

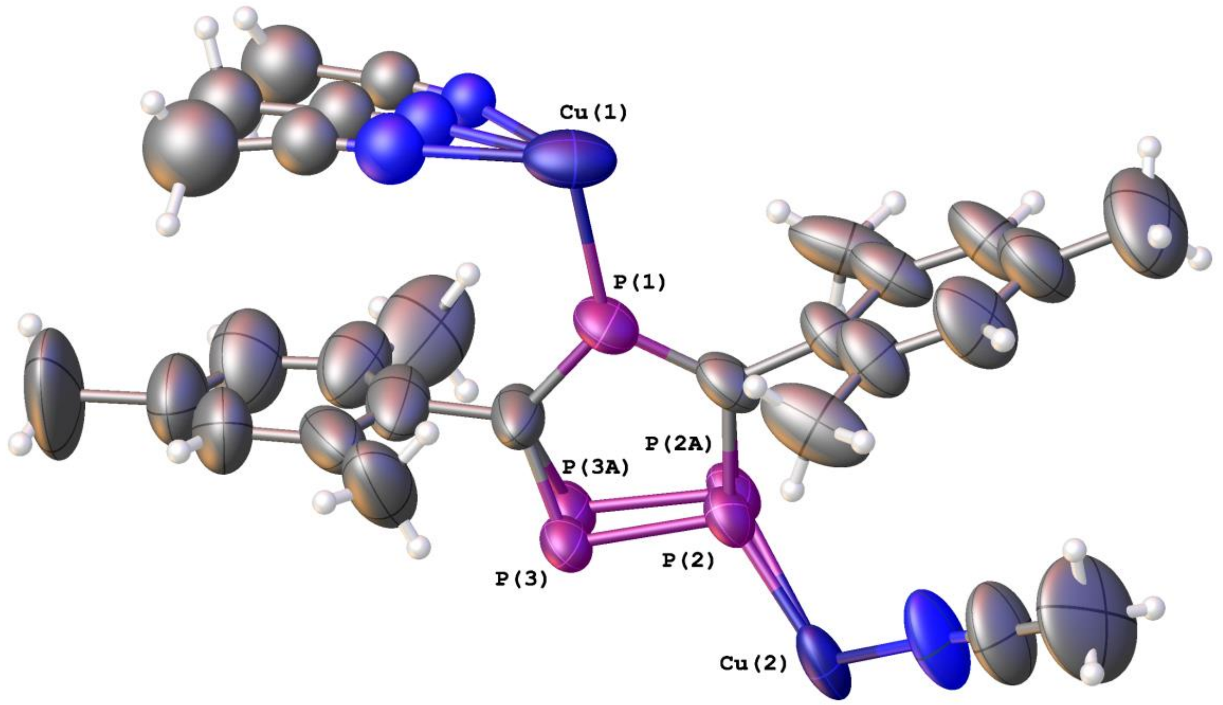

Fig. S 1: Section of the polymeric structure of **2**. The counter anion Cl^- (disordered over 6 positions) and solvated MeCN molecules (disordered over 2 positions) are not shown for clarity.

Table S 4: Selected geometric parameters for **2** (Å)

Cu1—N1B^{i}	1.83 (3)	Cu2—P2^{ii}	2.472 (6)
Cu1—N1B	1.83 (3)	Cu2—Cu2^{ii}	2.554 (3)
Cu1—N1A	2.07 (2)	Cu2—P2A	2.611 (10)
Cu1—N1A^{i}	2.07 (2)	P1—C1	1.718 (14)
Cu1—P1^{i}	2.243 (3)	P2—C1	1.771 (12)
Cu1—P1	2.243 (3)	P2—P3	2.079 (5)
Cu1—N1	2.36 (4)	P2—Cu2^{ii}	2.472 (6)
Cu1—N1^{i}	2.36 (4)	P3—Cu2^{iv}	2.277 (4)
Cu2—N2	1.956 (11)	P2A—C1	1.860 (14)
Cu2—P2A^{ii}	2.027 (10)	P2A—Cu2^{ii}	2.027 (10)
Cu2—P2	2.195 (6)	P2A—P3A	2.083 (11)
Cu2—P3^{iii}	2.277 (4)	P3A—Cu2^{iv}	2.317 (6)
Cu2—P3A^{iii}	2.317 (6)		

Symmetry code(s): (i) *x*, -*y*+1/2, -*z*+1/4; (ii) -*x*, -*y*, *z*; (iii) -*y*, *x*, -*z*; (iv) *y*, -*x*, -*z*.

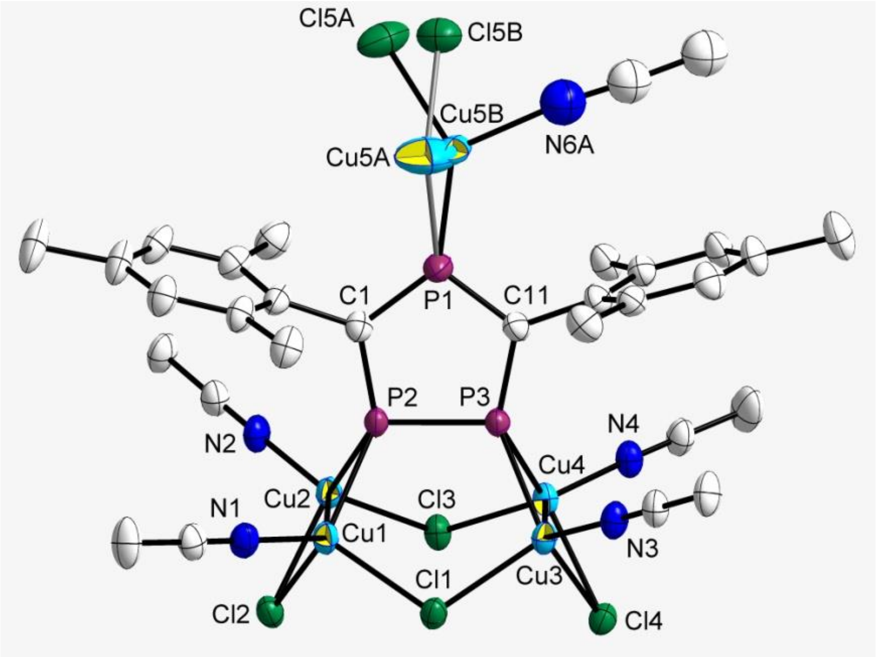

Fig. S 2: Section of the polymeric structure of **3**

Table S 5: Selected geometric parameters for **3** (Å)

Cu1—N1	1.972 (4)	Cu5A—Cl5A	2.338 (12)
Cu1—P2	2.2795 (13)	Cu5A—Cl5A^{i}	2.455 (12)
Cu1—Cl1	2.3692 (13)	Cl5A—Cu5B	2.407 (5)
Cu1—Cl2	2.4056 (13)	Cl5A—Cu5B^{i}	2.425 (6)
Cu2—N2	1.960 (4)	Cl5A—Cu5A^{i}	2.455 (12)
Cu2—P2	2.2900 (13)	Cu5B—N6A	2.084 (13)
Cu2—Cl2	2.3686 (12)	Cu5B—Cl5B	2.107 (6)
Cu2—Cl3	2.3733 (12)	Cu5B—P1	2.174 (4)
Cu3—N3	1.962 (4)	Cu5B—Cl5B^{i}	2.296 (6)
Cu3—P3	2.2964 (13)	Cu5B—Cl5A^{i}	2.425 (6)
Cu3—Cl4	2.3707 (12)	Cl5B—Cu5A^{i}	2.132 (13)
Cu3—Cl1	2.3823 (12)	Cl5B—Cu5B^{i}	2.296 (6)
Cu4—N4	1.964 (4)	N6A—C35A	1.196 (18)
Cu4—P3	2.2742 (12)	P1—C1	1.721 (5)
Cu4—Cl3	2.3541 (13)	P1—C11	1.721 (5)
Cu4—Cl4	2.4251 (13)	P2—C1	1.737 (5)
Cu5A—P1	2.125 (10)	P2—P3	2.0862 (14)
Cu5A—Cl5B^{i}	2.132 (13)	P3—C11	1.742 (5)
Cu5A—Cl5B	2.231 (11)		

Symmetry code(s): (i) $-x+2$, $-y+1$, $-z$.

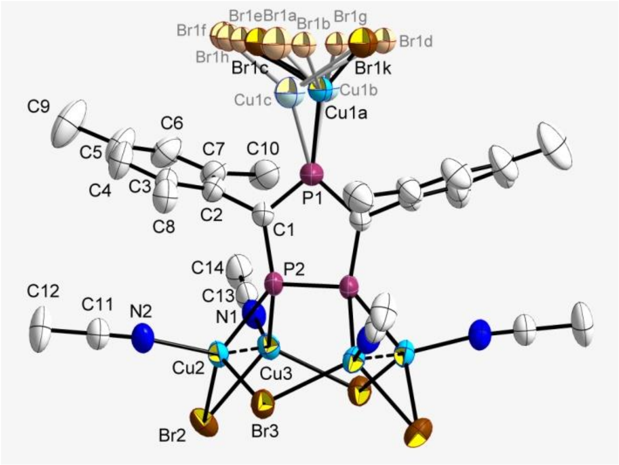

Fig. S 3: Section of the polymeric structure of **4**

Table S 6: Selected geometric parameters for **4** (Å)

Cu1A—Br1B	2.423 (11)	C4—C5	1.379 (8)
Cu1A—Br1E^{i}	2.468 (7)	C5—C6	1.368 (9)
Cu1A—Br1C^{i}	2.688 (7)	C5—C9	1.535 (8)
Br1C—Cu1A^{i}	2.688 (7)	C6—C7	1.391 (7)
Br1C—Cu1A^{ii}	2.688 (7)	C7—C10	1.506 (7)
Br1E—Cu1A^{iii}	2.370 (7)	C11—N2	1.125 (6)
Br1E—Cu1A^{ii}	2.468 (7)	C11—C12	1.445 (7)
Br1E—Cu1A^{i}	2.468 (7)	C13—N1	1.131 (6)
Br1B—Cu1A^{iii}	2.423 (11)	C13—C14	1.454 (6)
Cu1B—P1	2.159 (4)	P1—C1^{ii}	1.725 (5)
Cu1B—Br1A^{i}	2.429 (10)	P1—Cu1B^{ii}	2.159 (4)
Cu1B—Br1K	2.495 (9)	P1—Cu1A^{ii}	2.198 (4)
Cu1B—Br1K^{i}	2.632 (9)	P1—Cu1C^{ii}	2.328 (9)
Br1A—Cu1B^{i}	2.429 (10)	P2—P2^{ii}	2.092 (2)
Br1A—Cu1B^{ii}	2.429 (10)	K1—O1	2.600 (10)
Br1F—Cu1B^{ii}	2.307 (8)	K1—O2	2.82 (6)
Br1F—Cu1B^{i}	2.307 (8)	K1—N4^{iv}	3.110 (17)
Br1K—Cu1B^{iii}	2.495 (9)	K1—N4	3.110 (17)
Br1K—Cu1B^{i}	2.632 (9)	K1—Br3^{ii}	3.2102 (12)
Br1K—Cu1B^{ii}	2.632 (9)	K1—Br2^{ii}	3.3438 (8)
Cu1C—P1	2.328 (9)	N3—C19^{v}	1.297 (17)
Cu1C—Br1D	2.679 (14)	N3—C19	1.298 (16)
Br1D—Cu1C^{iii}	2.679 (14)	C19—C20	1.57 (3)
Cu2—N2	1.975 (4)	C19—C19^{v}	1.84 (3)
Cu2—P2	2.2802 (12)	O1—C18	1.405 (19)
Cu2—Br3	2.4801 (8)	O1—C15	1.45 (2)
Cu2—Br2	2.5287 (8)	O1—K1^{iv}	2.600 (10)

Cu2—Cu3	2.5477 (9)	C15—C16	1.58 (2)
Cu2—K1	3.9192 (15)	C15—K1^{iv}	3.442 (18)
Cu3—N1	1.968 (4)	C16—C17	1.57 (2)
Cu3—P2	2.2938 (13)	C17—C18	1.59 (2)
Cu3—Br2	2.4907 (8)	C18—K1^{iv}	3.380 (14)
Cu3—Br3^{ii}	2.5047 (8)	O2—C26	1.49 (3)
Cu3—K1	3.9334 (15)	O2—C23	1.51 (3)
Br3—Cu3^{ii}	2.5046 (8)	O2—K1^{iv}	2.74 (5)
Br3—K1	3.2102 (12)	C23—C24	1.57 (2)
Br2—K1	3.3438 (8)	C24—C25	1.58 (2)
C1—C2	1.491 (6)	C25—C26	1.58 (2)
C1—P1	1.725 (5)	N4—C21	1.21 (4)
C1—P2	1.735 (5)	N4—K1^{iv}	3.110 (17)
C2—C3	1.403 (7)	C21—C22	1.49 (5)
C2—C7	1.411 (7)	N4A—C21A	1.19 (7)
C3—C4	1.395 (7)	C21A—C22A	1.55 (8)
C3—C8	1.512 (7)		

Symmetry code(s): (i) -*x*+1, -*y*+1, -*z*+1; (ii) -*x*+1, *y*, -*z*+1; (iii) *x*, -*y*+1, *z*; (iv) -*x*+1, -*y*, -*z*+1; (v) -*x*+2, *y*, -*z*+2.

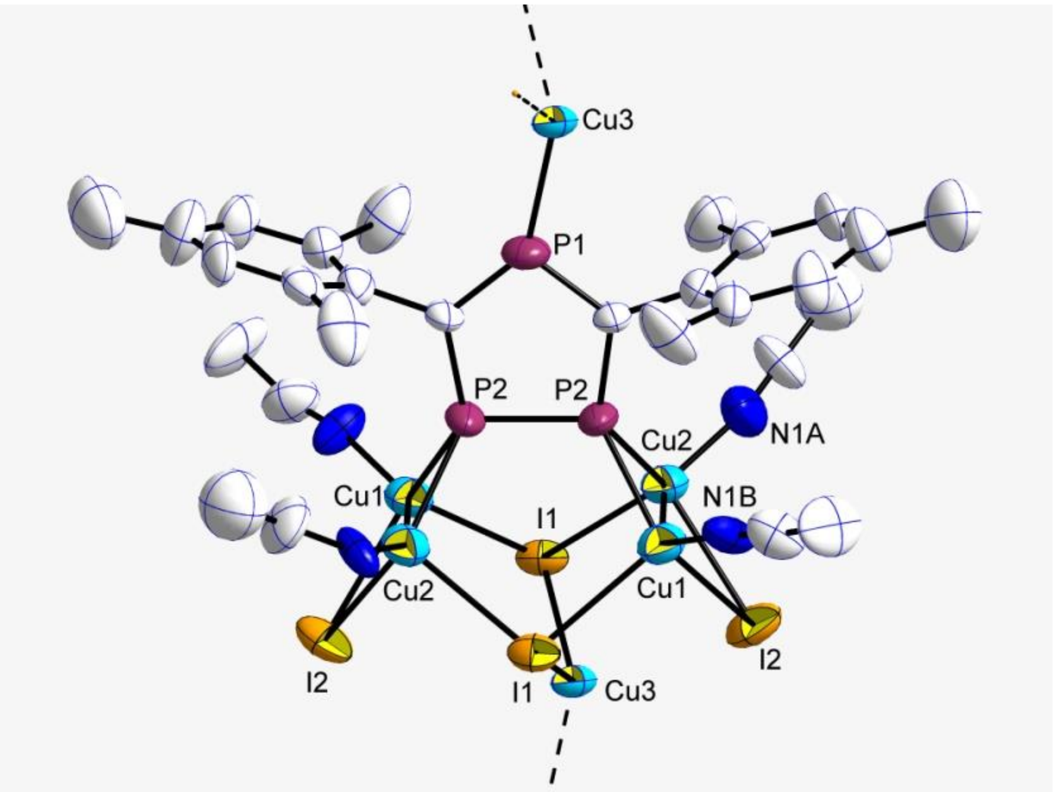

Fig. S 4: Section of the polymeric structure of **5**

Table S 7: Selected geometric parameters for **5** (Å)

Cu1—Cu2	2.541 (4)	Cu3—I2	2.978 (12)
Cu1—N1B	1.94 (2)	Cu3—P1^{iv}	2.249 (6)
Cu1—I1^{ii}	2.673 (4)	I1—Cu1^{ii}	2.673 (4)
Cu1—I2	2.677 (3)	I1—Cu3^{ii}	2.59 (3)
Cu1—P2	2.277 (5)	P1—Cu3^{v}	2.249 (6)
N1A—Cu1^{iii}	2.28 (3)	P1—Cu3^{vi}	2.249 (6)
N1A—Cu2	2.02 (4)	P1—Cu3^{vii}	2.249 (6)
Cu2—N1B^{iii}	2.03 (2)	P1—Cu3^{viii}	2.249 (6)
Cu2—I1	2.676 (4)	P1—C1^{ii}	1.706 (16)
Cu2—I2	2.678 (3)	P1—C1	1.706 (16)
Cu2—P2	2.319 (6)	P1—C1^{iii}	1.706 (16)
Cu2—P2^{iii}	2.529 (6)	P2—C1	1.738 (15)
Cu3—I1^{ii}	2.59 (3)	P2—P2^{ii}	2.099 (9)
Cu3—I1	2.67 (3)	P2—Cu1^{iii}	2.697 (6)

Symmetry code(s): (i) *y*, -*x*+3/2, *z*; (ii) -*x*+3/2, -*y*+3/2, *z*; (iii) -*y*+3/2, *x*, *z*; (iv) *x*, *y*, *z*-1; (v) *x*, *y*, *z*+1; (vi) -*y*+3/2, *x*, *z*+1; (vii) *y*, -*x*+3/2, *z*+1; (viii) -*x*+3/2, -*y*+3/2, *z*+1.

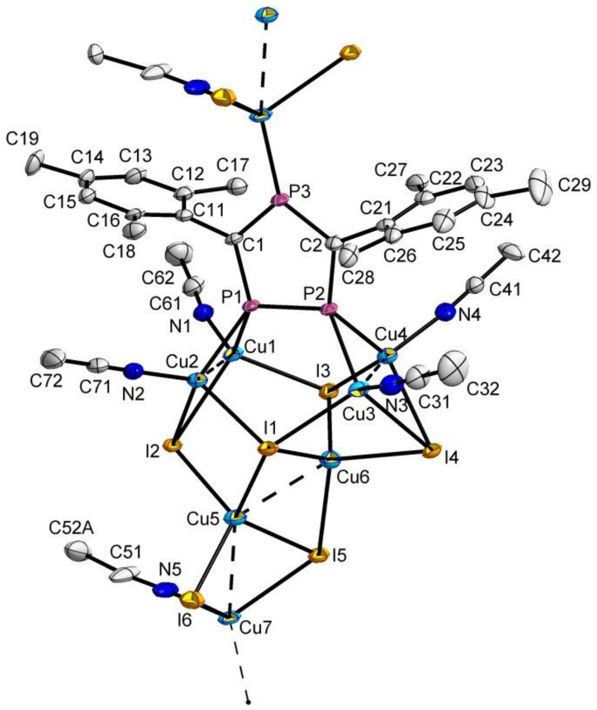

Fig. S 5: Section of the polymeric structure of **6**

Table S 8: Selected geometric parameters for **6** (Å)

P1—P2	2.101 (3)	Cu3—I4	2.6997 (13)
P1—C1	1.731 (7)	Cu3—Cu4	2.5229 (19)
P2—C2	1.740 (8)	Cu3—Cu6	2.9783 (19)
P3—Cu7^{i}	2.214 (2)	Cu4—P2	2.319 (2)
P3—C1	1.726 (9)	Cu4—N4	1.961 (8)
P3—C2	1.740 (8)	Cu4—I3	2.6770 (15)
N1—C61	1.163 (12)	Cu4—I4	2.7529 (13)
N2—C71	1.138 (12)	Cu4—Cu6	2.9091 (19)
N3—C31	1.136 (13)	Cu5—I1	2.7423 (15)
N4—C41	1.150 (12)	Cu5—I2	2.7004 (14)
N5—C51	1.110 (14)	Cu5—I5	2.6437 (15)
Cu1—P1	2.289 (2)	Cu5—I6	2.6174 (15)
Cu1—N1	1.964 (7)	Cu5—Cu6	2.759 (2)
Cu1—I2	2.7070 (14)	Cu5—Cu7	2.7347 (18)
Cu1—I3	2.6363 (13)	Cu6—I1	2.7022 (16)
Cu1—Cu2	2.5225 (19)	Cu6—I3	2.6484 (15)
Cu2—P1	2.289 (2)	Cu6—I4	2.6978 (15)
Cu2—N2	1.952 (8)	Cu6—I5	2.6417 (14)
Cu2—I1	2.7278 (13)	Cu7—P3^{ii}	2.214 (2)
Cu2—I2	2.6736 (14)	Cu7—N5	2.046 (8)
Cu3—P2	2.285 (2)	Cu7—I5	2.6977 (15)
Cu3—N3	1.971 (9)	Cu7—I6	2.6490 (16)
Cu3—I1	2.6760 (15)		

Symmetry code(s): (i) *x*-1, *y*+1, *z*; (ii) *x*+1, *y*-1, *z*.

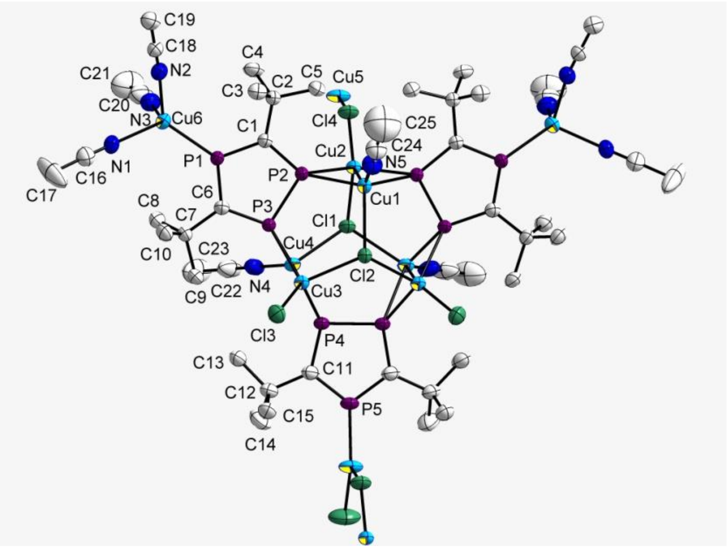

Fig. S 6: Section of the polymeric structure of **7**

Table S 9: Selected geometric parameters for **7** (Å)

C1—P1	1.731 (4)	Cl4—Cu5	2.300 (2)
C1—P2	1.737 (4)	Cl5A—Cu5	2.142 (9)
C1—C2	1.545 (5)	Cl5B—Cu5	2.281 (9)
C2—C3	1.519 (6)	Cu1—N5	1.858 (13)
C2—C5	1.520 (5)	Cu1—N3S	2.190 (18)
C2—C4	1.529 (6)	Cu1—P2^{i}	2.3250 (11)
C6—C7	1.542 (5)	Cu1—P2	2.3250 (11)
C6—P3	1.736 (4)	Cu1—Cu2	2.4961 (14)
C6—P1	1.742 (4)	Cu2—P2	2.3612 (11)
C7—C8	1.520 (6)	Cu2—P2^{i}	2.3612 (11)
C7—C9	1.527 (6)	Cu3—P3	2.3493 (10)
C7—C10	1.533 (6)	Cu3—P4	2.3566 (11)
C11—C12	1.542 (6)	Cu3—Cu4	2.4823 (9)
C11—P5	1.733 (4)	Cu4—N4	1.972 (4)
C11—P4	1.737 (4)	Cu4—P4	2.3207 (10)
C12—C14	1.511 (7)	Cu4—P3	2.3449 (12)
C12—C13	1.524 (6)	Cu5—P5^{ii}	2.1908 (15)
C12—C15	1.531 (7)	Cu5—Cl5B^{i}	2.281 (9)
Cl1—Cu2	2.4085 (16)	Cu6—N1	2.020 (4)
Cl1—Cu4	2.4159 (9)	Cu6—N3	2.053 (5)
Cl1—Cu4^{i}	2.4160 (9)	Cu6—N2	2.061 (4)
Cl2—Cu1	2.3897 (14)	Cu6—P1	2.2317 (10)
Cl2—Cu3	2.4409 (9)	P2—P3	2.0966 (13)
Cl2—Cu3^{i}	2.4410 (9)	P4—P4^{i}	2.0964 (19)
Cl3—Cu3	2.2305 (11)	P5—C11^{i}	1.733 (4)
Cl4—Cu2	2.2791 (16)	P5—Cu5^{iii}	2.1908 (15)

Symmetry code(s): (i) *x*, -*y*+1/2, *z*; (ii) *x*+1, *y*, *z*; (iii) *x*-1, *y*, *z*.

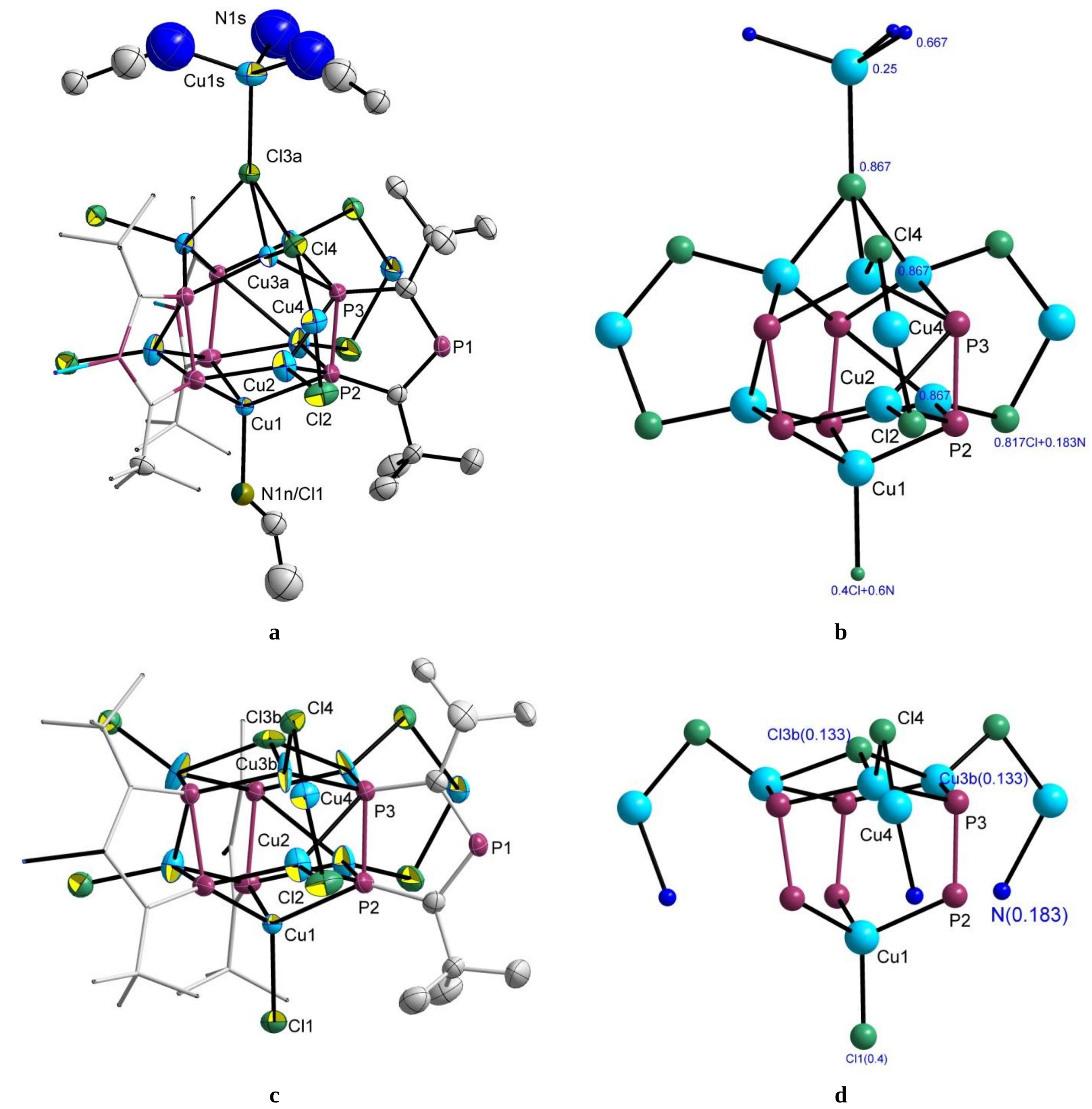

Fig. S 7: Section of the polymeric structure of **8**: (a) Major (0.867) and (c) minor (0.133) part in the disordered structure of **8** and occupation factors of the disordered atoms in major (b) and minor (d) parts, respectively. The structure **8** cannot be represented as an overlap of only two alternative cores according to occupancy factors (compare with 9). The coordinated MeCN molecules have larger occupancies as the corresponding Cu1s ion. Therefore, they are coordinated in the major part and solvated in the minor one.

Table S 10: Selected geometric parameters for **8** (Å)

Cu1S—N1S^{i}	2.22 (5)	Cu3A—P3^{iv}	2.314 (2)
Cu1S—N1S^{ii}	2.22 (5)	Cu3A—Cl4	2.354 (2)
Cu1S—N1S	2.22 (5)	Cu3A—Cl3A	2.466 (3)
Cu1S—Cu1S^{ii}	2.395 (17)	Cu3A—Cu3A^{iv}	2.808 (3)
Cu1S—Cu1S^{i}	2.395 (17)	Cu3A—Cu3A^{v}	2.808 (3)
Cu1S—Cu1S^{iii}	2.395 (17)	Cl3A—Cu3A^{v}	2.466 (3)
Cu1S—Cl3A	2.522 (11)	Cl3A—Cu3A^{iv}	2.466 (3)
N1S—C1S	1.14 (7)	Cu3B—Cl4	2.026 (9)

N1S—Cu1S^{i}	2.22 (5)	Cu3B—P3	2.325 (9)
N1S—Cu1S^{ii}	2.22 (5)	Cu3B—P3^{iv}	2.335 (9)
C1S—C2S	1.46 (5)	Cu3B—Cl3B	2.367 (18)
Cl1—Cu1	2.216 (6)	Cl3B—Cu3B^{v}	2.367 (18)
C1N—C1N^{iv}	1.42 (8)	Cl3B—Cu3B^{iv}	2.367 (18)
C1N—C1N^{v}	1.42 (8)	Cu4—P1^{viii}	2.200 (2)
C1N—C2N	1.55 (3)	Cu4—Cl4	2.261 (2)
C2N—C2N^{vi}	1.00 (9)	P1—C6	1.725 (8)
C2N—C2N^{vii}	1.00 (9)	P1—C1	1.733 (8)
C2N—C2N^{v}	1.98 (14)	P1—Cu4^{ix}	2.200 (2)
C2N—C2N^{iv}	1.98 (14)	P2—C1	1.744 (7)
Cu1—P2	2.3528 (19)	P2—P3	2.108 (3)
Cu1—P2^{v}	2.3529 (19)	P2—Cu2^{v}	2.304 (3)
Cu1—P2^{iv}	2.3529 (19)	P3—C6	1.746 (7)
Cu1—Cu2^{iv}	2.6826 (17)	P3—Cu3A^{v}	2.314 (2)
Cu1—Cu2^{v}	2.6826 (17)	P3—Cu3B^{v}	2.335 (9)
Cu1—Cu2	2.6827 (17)	C1—C2	1.544 (10)
Cu2—Cl2	2.200 (3)	C2—C3	1.493 (13)
Cu2—P2	2.275 (3)	C2—C4	1.515 (13)
Cu2—P2^{iv}	2.304 (3)	C2—C5	1.542 (15)
Cu2—P3	2.655 (2)	C6—C7	1.539 (10)
Cu2—Cu3A	2.778 (2)	C7—C10	1.533 (11)
Cl2—Cu4	2.299 (3)	C7—C9	1.545 (11)
Cu3A—P3	2.302 (2)	C7—C8	1.545 (11)

Symmetry code(s): (i) -*x*+3/2, *y*, -*z*+3/2; (ii) -*x*+3/2, -*y*+3/2, *z*; (iii) *x*, -*y*+3/2, -*z*+3/2; (iv) *z*, *x*, *y*; (v) *y*, *z*, *x*; (vi) *y*, -*z*+2, -*x*+2; (vii) -*z*+2, *x*, -*y*+2; (viii) -*z*+2, -*y*+3/2, *x*; (ix) *z*, -*y*+3/2, -*x*+2.

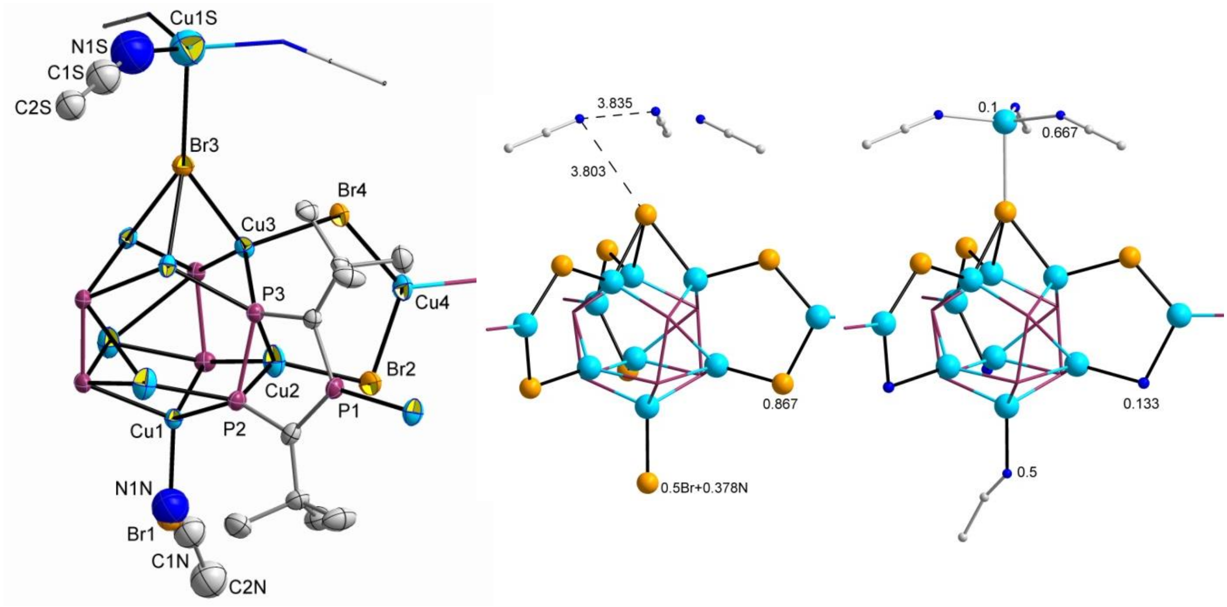

a **b** **c**

Fig. S 8: Section of the polymeric structure of **9**: (a) Numerating scheme, (b) major (0.867) and (c) minor (0.133) part in the disordered structure of **9**. All positions of Cu are fully occupied, the environment of Cu anions in case of the disorder is completed by Br or MeCN molecules. The latter is localized in the position Br1 and not localized in position Br2 due to its smaller contribution. The coordinated MeCN molecules have larger occupancies as the corresponding Cu1s ion. Therefore, they are coordinated in minor part and solvated in major one.

Table S 5: Selected geometric parameters for **9** (Å)

Cu1S—N1S^{i}	2.23 (4)	Cu2—Cu3	2.7828 (17)
Cu1S—N1S^{ii}	2.23 (4)	Br2—Cu4	2.3921 (14)
Cu1S—N1S	2.23 (4)	Cu3—P3	2.3228 (17)
Cu1S—Br3	2.80 (4)	Cu3—P3^{iv}	2.3327 (16)
N1S—C1S	1.19 (5)	Cu3—Br4	2.4370 (11)
N1S—Cu1S^{i}	2.23 (4)	Cu3—Br3	2.5728 (12)
N1S—Cu1S^{ii}	2.23 (4)	Cu3—Cu3^{iv}	2.7531 (15)
C1S—C2S	1.35 (4)	Cu3—Cu3^{iii}	2.7532 (15)
Br1—Cu1	2.402 (5)	Br3—Cu3^{iii}	2.5728 (12)
N1N—C1N	1.16 (6)	Br3—Cu3^{iv}	2.5728 (12)
N1N—C1N^{iii}	1.16 (6)	Cu4—P1^{vii}	2.2013 (18)
N1N—C1N^{iv}	1.16 (6)	Cu4—Br4	2.3870 (12)
N1N—Cu1	2.100 (14)	P1—C1	1.720 (7)
C1N—C1N^{iii}	1.52 (11)	P1—C6	1.725 (6)
C1N—C1N^{iv}	1.52 (11)	P1—Cu4^{viii}	2.2015 (18)
C1N—C2N	1.54 (3)	P2—C1	1.754 (6)
C2N—C2N^{v}	1.35 (10)	P2—P3	2.108 (2)
C2N—C2N^{vi}	1.35 (10)	P2—Cu2^{iii}	2.2878 (19)
Cu1—P2	2.3587 (15)	P3—C6	1.744 (6)
Cu1—P2^{iii}	2.3588 (15)	P3—Cu3^{iii}	2.3328 (16)
Cu1—P2^{iv}	2.3588 (15)	C1—C2	1.529 (9)

Cu1—Cu2	2.6939 (14)	C2—C3	1.509 (10)
Cu1—Cu2^{iii}	2.6939 (14)	C2—C4	1.527 (11)
Cu1—Cu2^{iv}	2.6939 (14)	C2—C5	1.536 (11)
Cu2—P2^{iv}	2.2878 (19)	C6—C7	1.535 (8)
Cu2—P2	2.3324 (19)	C7—C8	1.521 (9)
Cu2—Br2	2.3384 (14)	C7—C10	1.533 (9)
Cu2—P3	2.6195 (19)	C7—C9	1.561 (10)

Symmetry code(s): (i) -*x*+1/2, *y*, -*z*+1/2; (ii) -*x*+1/2, -*y*+1/2, *z*; (iii) *y*, *z*, *x*; (iv) *z*, *x*, *y*; (v) -*z*, *x*, -*y*; (vi) *y*, -*z*, -*x*; (vii) -*z*, -*y*+1/2, *x*; (viii) *z*, -*y*+1/2, -*x*.

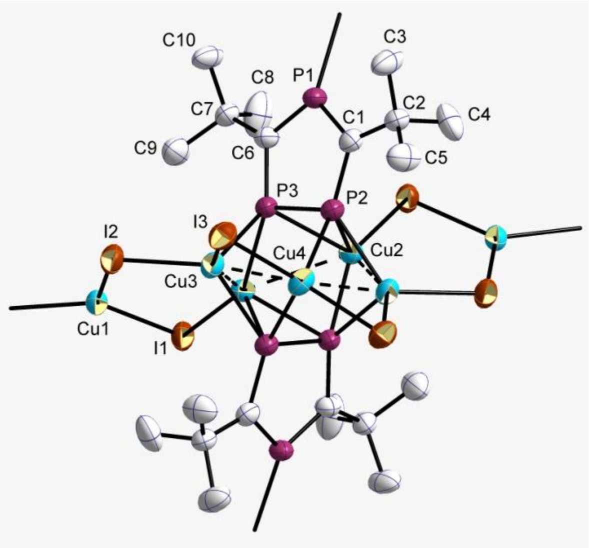

Fig. S 9: Section of the polymeric structure of **10.**

Table S 6: Selected geometric parameters for **10** (Å)

I1—Cu2^{i}	2.4869 (18)	C7—C8	1.52 (2)
I1—Cu1	2.5485 (18)	C7—C9	1.540 (18)
I2—Cu3	2.5399 (18)	K1—C24A^{iv}	3.45 (6)
I2—Cu1	2.545 (2)	K1—C11^{iv}	3.49 (4)
I3—Cu4	2.5692 (13)	O1—C11	1.31 (4)
I3—Cu3	2.6503 (19)	O1—C14	1.60 (5)
Cu1—P1^{ii}	2.215 (3)	C11—C12	1.33 (5)
Cu2—P3^{i}	2.254 (3)	C12—C13	1.48 (4)
Cu2—P2	2.471 (3)	C13—C14	1.70 (6)
Cu2—P3	2.484 (3)	O1A—C14A	1.33 (5)
Cu2—I1^{i}	2.4869 (18)	O1A—C11A	1.51 (5)
Cu2—Cu3^{i}	2.663 (2)	C11A—C12A	1.20 (6)
Cu2—Cu2^{i}	2.793 (3)	C12A—C13A	1.57 (5)
Cu3—P2^{i}	2.398 (3)	C13A—C14A	1.32 (6)
Cu3—Cu4	2.540 (2)	O2—C24	1.43 (4)

Cu3—P3	2.559 (3)	O2—C21	1.50 (4)
Cu3—Cu2^{i}	2.663 (2)	C21—C22	1.49 (5)
Cu4—P2	2.463 (3)	C22—C23	1.528 (19)
Cu4—P2^{i}	2.463 (3)	C23—C24	1.535 (19)
Cu4—Cu3^{i}	2.540 (2)	O2A—C24A	1.45 (9)
Cu4—I3^{i}	2.5692 (13)	O2A—C21A	1.66 (7)
P1—C6	1.710 (11)	C21A—C22A	1.55 (2)
P1—C1	1.739 (12)	C22A—C23A	1.76 (7)
P1—Cu1^{iii}	2.215 (3)	C23A—C24A	1.54 (2)
P2—C1	1.751 (11)	O3—C34	1.42 (2)
P2—P3	2.120 (4)	O3—C31	1.45 (2)
P2—Cu3^{i}	2.398 (3)	C31—C32	1.49 (2)
P3—C6	1.743 (11)	C32—C33	1.54 (2)
P3—Cu2^{i}	2.254 (3)	C33—C34	1.46 (3)
C1—C2	1.525 (16)	O1S—C1S	1.37 (2)
C2—C3	1.497 (17)	O1S—C4S	1.38 (2)
C2—C5	1.515 (17)	C1S—C2S	1.52 (8)
C2—C4	1.522 (19)	C2S—C3S	1.50 (9)
C6—C7	1.545 (15)	C4S—C3S	1.53 (2)
C7—C10	1.514 (18)		
P1^{ii}—Cu1—I2	116.92 (11)	P2^{i}—Cu3—I3	110.87 (10)
P1^{ii}—Cu1—I1	127.43 (11)	I2—Cu3—I3	109.25 (7)
I2—Cu1—I1	115.64 (7)	P3—Cu3—I3	115.69 (9)
P3^{i}—Cu2—P2	97.82 (11)	P2—Cu4—P2^{i}	95.99 (15)
P3^{i}—Cu2—P3	98.29 (11)	P2—Cu4—I3	103.55 (7)
P2—Cu2—P3	50.67 (10)	P2^{i}—Cu4—I3	111.51 (7)
P3^{i}—Cu2—I1^{i}	142.20 (10)	P2—Cu4—I3^{i}	111.51 (7)
P2—Cu2—I1^{i}	116.35 (9)	P2^{i}—Cu4—I3^{i}	103.55 (7)
P3—Cu2—I1^{i}	115.42 (9)	I3—Cu4—I3^{i}	126.65 (11)
P2^{i}—Cu3—I2	121.49 (10)	P1—C1—P2	115.3 (7)
P2^{i}—Cu3—P3	91.89 (11)	P1—C6—P3	116.1 (6)
I2—Cu3—P3	106.91 (9)		

Symmetry code(s): (i) -*x*+2, *y*, -*z*+1/2; (ii) -*x*+3/2, -*y*+1/2, *z*+1/2; (iii) -*x*+3/2, -*y*+1/2, *z*-1/2; (iv) -*x*+2, -*y*, -*z*+1.

3. **Crystallographic problem in 2:** Quality of the diffraction experiment

3.1 Indexation of the diffraction pattern

It is obvious that

1) Primitive and Bravais unit cell are correct (no superstructural reflections, no multiplied axes, see Fig. S 10);

2) Most of the reflections belong to the reciprocal lattice (have integer *hkl*) (Fig. S 11);
3) There is no visible signs for twinning (Fig. S 10 – S 11).

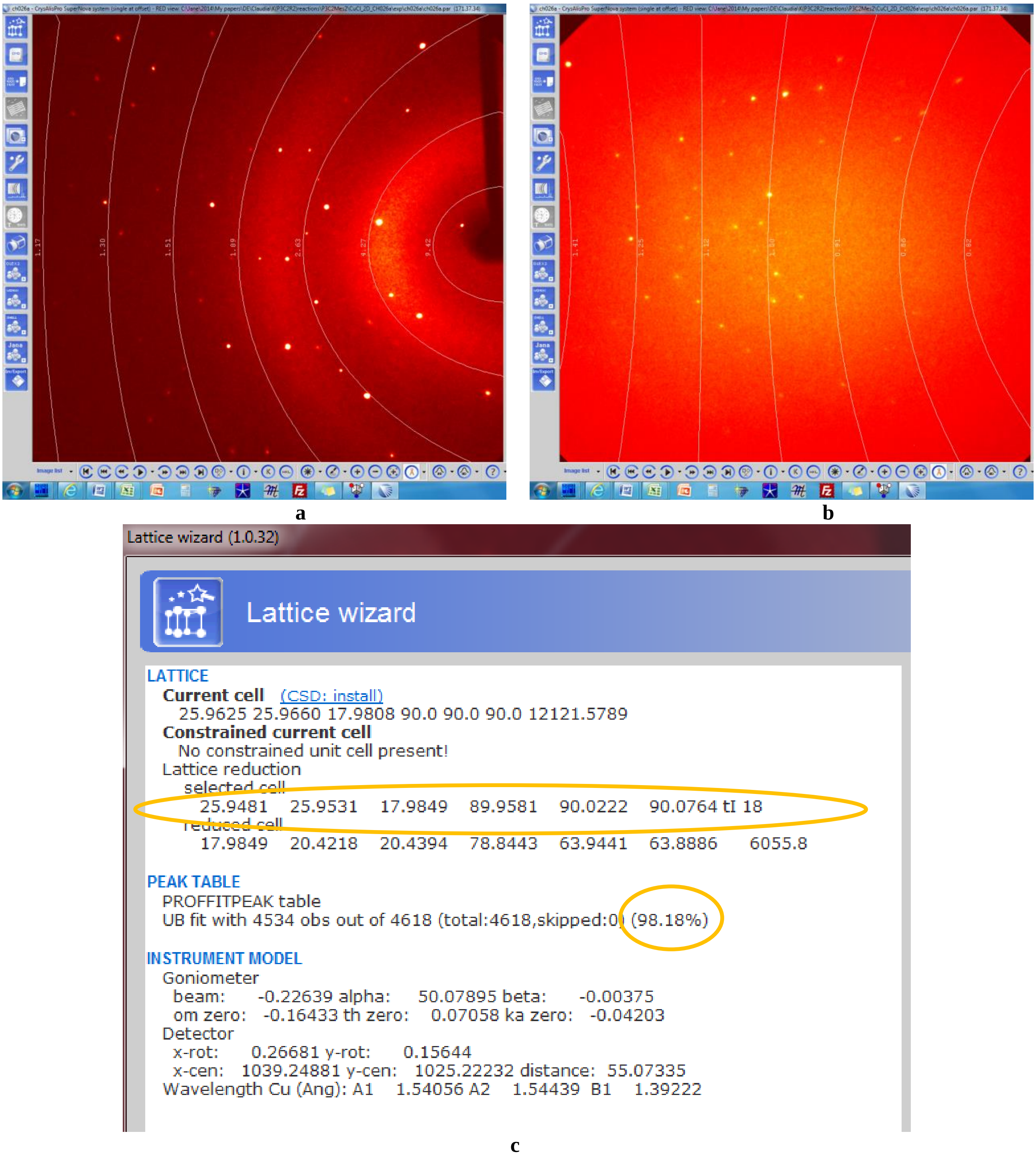

Fig. S 10: An example of a frame at low (a) and at high (b) resolution and (c) indexation in the chosen unit cell (*Crysalis Pro* software).

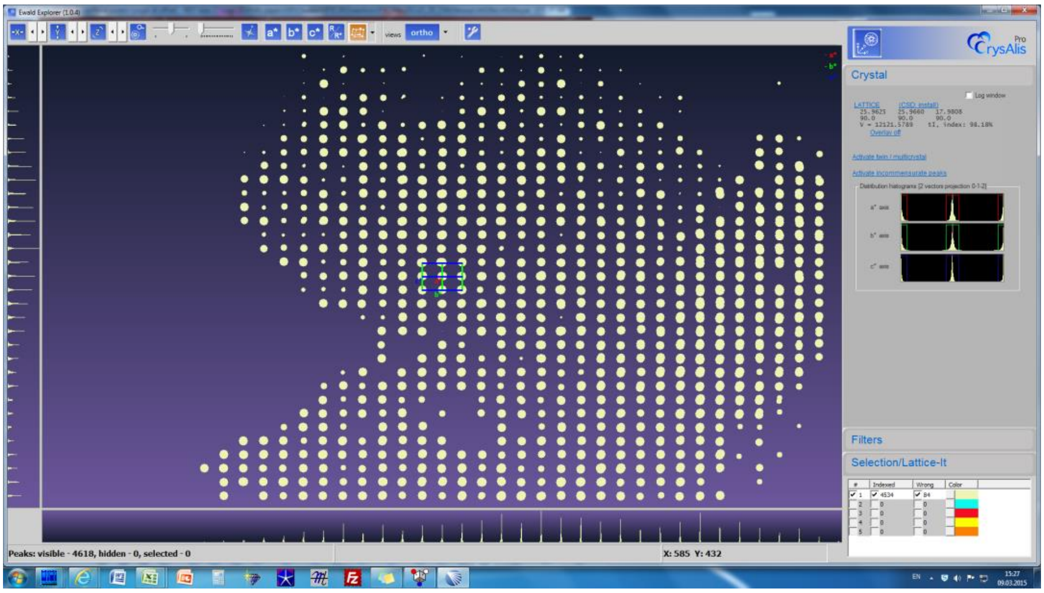

a

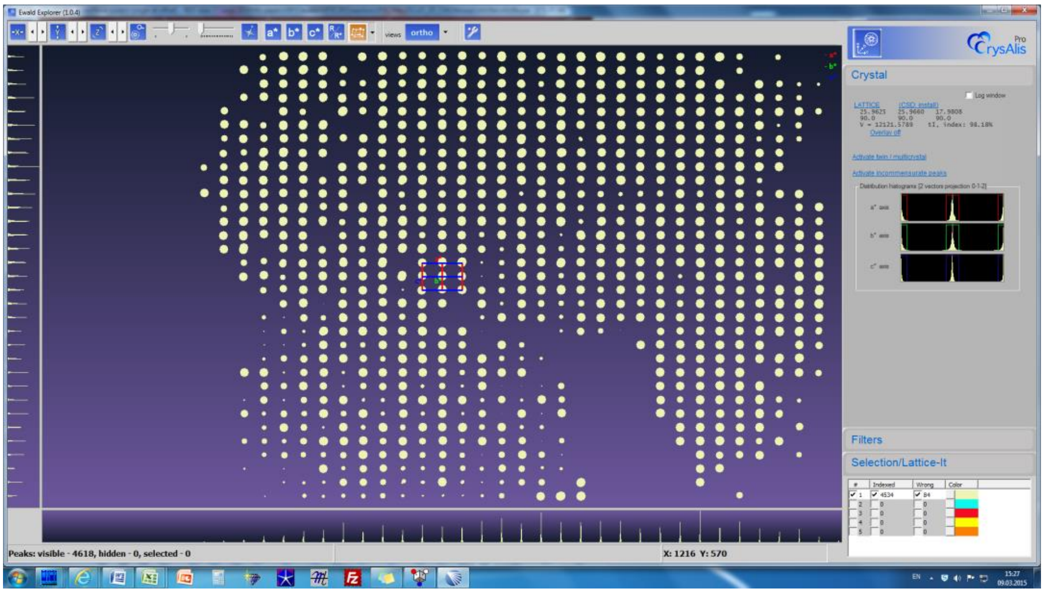

b

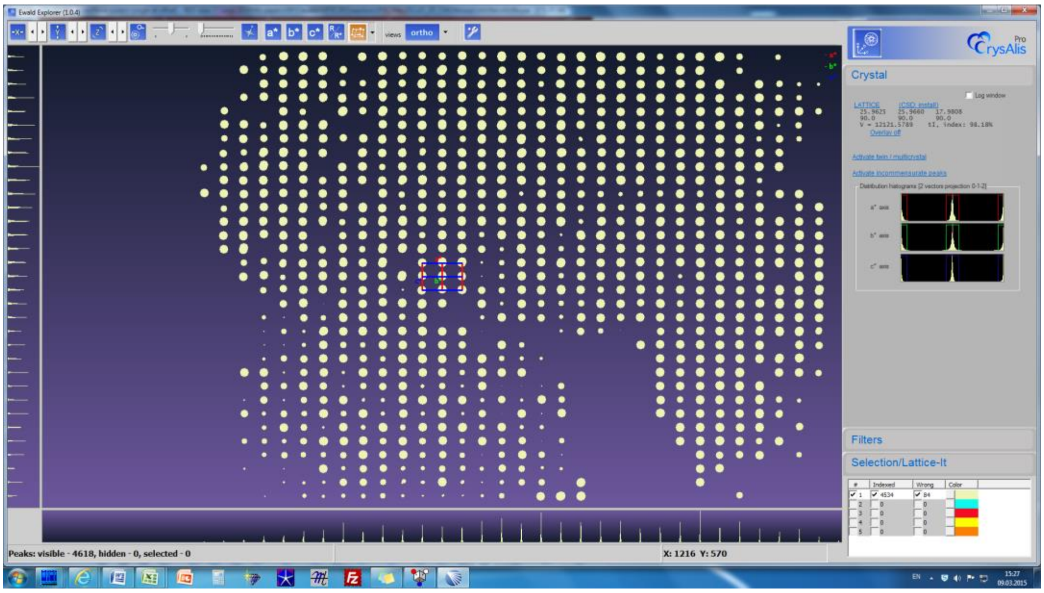

c

Fig. S 11: View along (a) ***a****, (b) ***b**** and (c) ***c**** directions (*Crysalis Pro* software)

3.2 *Intensity statistics visualization by means of Wilson plot*

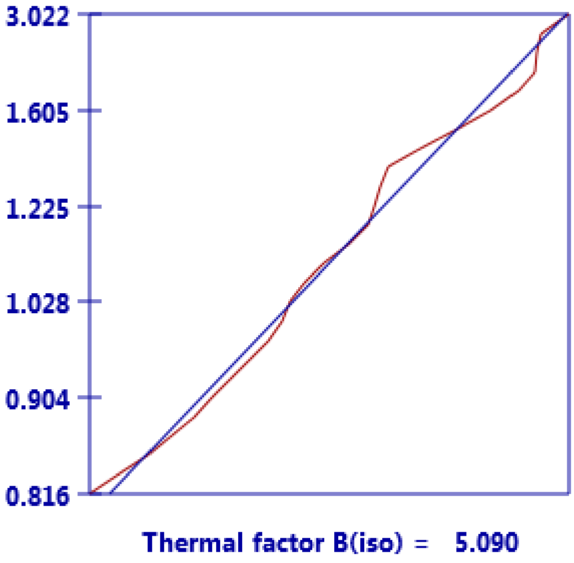

Fig. S 12: Wilson plot (*SIR* Software).

3.3 *Data analysis in XPREP*

```
Original cell in Angstroms and degrees:
   25.853   25.853   17.996    90.00    90.00    90.00
   25827 Reflections read from file tp.hkl; mean (I/sigma) =    5.56
Lattice exceptions:  P      A      B      C      I      F     Obv    Rev    All
N (total) =          0  12903  12876  12941  12986  19360  17163  17173  25827
N (int>3sigma) =     0   3762   3796   3914      4   5736   5061   5059   7609
Mean intensity =   0.0    4.2    4.3    4.4    0.1    4.3    4.4    4.4    4.3
Mean int/sigma =   0.0    5.7    5.8    6.0    0.3    5.8    5.8    5.8    5.7
-------------------------------------------------------------------------------
Search for higher metric symmetry
Identical indices and Friedel opposites combined before calculating R(sym)
------------------------------------------------------------------------------
Option A: FOM = 0.000 deg.   TETRAGONAL   I-lattice   R(sym) = 0.027 [   4785]
Cell:    25.853  25.853  17.996   90.00   90.00   90.00    Volume:     12027.65
Matrix: 1.0000  0.0000  0.0000  0.0000  1.0000  0.0000  0.0000  0.0000  1.0000
-------------------------------------------------------------------------------
SPACE GROUP DETERMINATION
Lattice exceptions:  P      A      B      C      I      F     Obv    Rev    All

N (total) =          0  12903  12876  12941  12986  19360  17163  17173  25827
N (int>3sigma) =     0   3762   3796   3914      4   5736   5061   5059   7609
Mean intensity =   0.0    4.2    4.3    4.4    0.1    4.3    4.4    4.4    4.3
Mean int/sigma =   0.0    5.7    5.8    6.0    0.3    5.8    5.8    5.8    5.7

Crystal system T and Lattice type I selected

Mean |E*E-1| = 0.885 [expected .968 centrosym and .736 non-centrosym]

Chiral flag NOT set

Systematic absence exceptions:
        41/43  a--   -c-   --d
N          5   226   352   232
N I>3s     0    97   229     0
<I>      0.1  22.0  23.8   0.1
<I/s>    0.2  12.6  19.1   0.3
```

Identical indices and Friedel opposites combined before calculating R(sym)

Option	Space Group	No.	Type	Axes	CSD	R(sym)	N(eq)	Syst. Abs.	CFOM
[A]	I-42d	#122	non-cen	1	36	0.030	7526	0.3 / 5.7	6.75
[B]	I4(1)md	#109	non-cen	1	6	0.030	7526	0.3 / 5.7	18.34

Option [A] chosen

INTENSITY STATISTICS FOR DATASET # 1 tp.hkl

Resolution	#Data	#Theory	%Complete	Redundancy	Mean I	Mean I/s	R(int)	Rsigma
Inf - 2.32	168	171	98.2	3.48	76.4	75.94	0.0181	0.0093
2.32 - 1.81	173	173	100.0	3.46	39.6	37.12	0.0286	0.0187
1.81 - 1.56	177	177	100.0	3.12	21.7	22.17	0.0381	0.0371
1.56 - 1.41	178	178	100.0	2.94	9.3	13.02	0.0494	0.0746
1.41 - 1.30	173	173	100.0	6.27	5.4	44.30	0.0529	0.0219
1.30 - 1.22	174	174	100.0	6.33	4.8	41.20	0.0590	0.0228
1.22 - 1.16	170	170	100.0	5.44	4.3	33.83	0.0612	0.0275
1.16 - 1.10	197	197	100.0	4.27	3.3	27.09	0.0362	0.0361
1.10 - 1.05	205	205	100.0	4.25	1.9	16.56	0.0465	0.0608
1.05 - 1.01	186	186	100.0	4.07	1.6	14.30	0.0521	0.0725
1.01 - 0.98	170	170	100.0	4.03	1.9	16.75	0.0467	0.0605
0.98 - 0.95	189	189	100.0	3.77	1.2	11.07	0.0593	0.0944
0.95 - 0.92	205	205	100.0	3.77	1.0	9.64	0.0621	0.1058
0.92 - 0.89	242	242	100.0	3.51	0.7	7.81	0.0744	0.1324
0.89 - 0.87	177	177	100.0	3.26	0.6	6.79	0.0783	0.1543
0.87 - 0.85	197	200	98.5	3.05	0.4	5.63	0.0953	0.1846
0.85 - 0.83	195	203	96.1	2.57	0.3	3.89	0.1018	0.2838
0.83 - 0.80	135	199	67.8	1.33	0.2	3.28	0.1006	0.3460
0.90 - 0.80	874	949	92.1	2.70	0.5	5.48	0.0848	0.1902
Inf - 0.80	3311	3389	97.7	3.79	9.1	21.05	0.0327	0.0260

Merged [A], lowest resolution = 9.73 Angstroms, 281 outliers downweighted

3.4 Space Group Determination and Refinement

In the tetragonal $I\bar{4}2d$ space group (best R(sym)=0.03, current model) the quality factors are R_1=0.095, wR_2=0.248 (for observed reflections), GooF=1.043 providing that racemic twinning batch refined to 0.20(10). In this space group no other twinning by merohedry is possible. All atoms have high displacement parameters, and the splitting of their positions does not improve the geometry of the complex.

The attempts to find another better solution failed with both SHEXL and SIR2014. The attempts to solve the structure in $I4_1md$ did not return a more reasonable model. It is also in contradiction with the refined twinning batch. The attempts to solve the structure in the *t-subgroup* $I\bar{4}$ and $I2_12_12_1$ with corresponding twinning models did not yield better quality factors.

We assumed also the possibility of an orthorhombic-to-tetragonal twinning from $Fdd2$ (with UC a=36.7180, b=36.6889, c=17.9812) to $I\bar{4}2d$ with {0 1 0, -1 0 0, 0 0 -1} twinning element. The disorder does not disappear.

In addition, a second X-ray diffraction experiment of crystals of **2**, crystallized from a different sample, shows the same phenomenon as described above.

4. Diffuse scattering in 5

The diffraction pattern of **5** shows quite strong diffuse scattering (Fig. S 13) that is visible even during the routine diffraction experiment. The diffraction pattern in the reciprocal space reconstructed with CrysAlisPro (Agilent Technologies) software shows that in addition to the Braggs peaks, two types of diffuse spots appear.

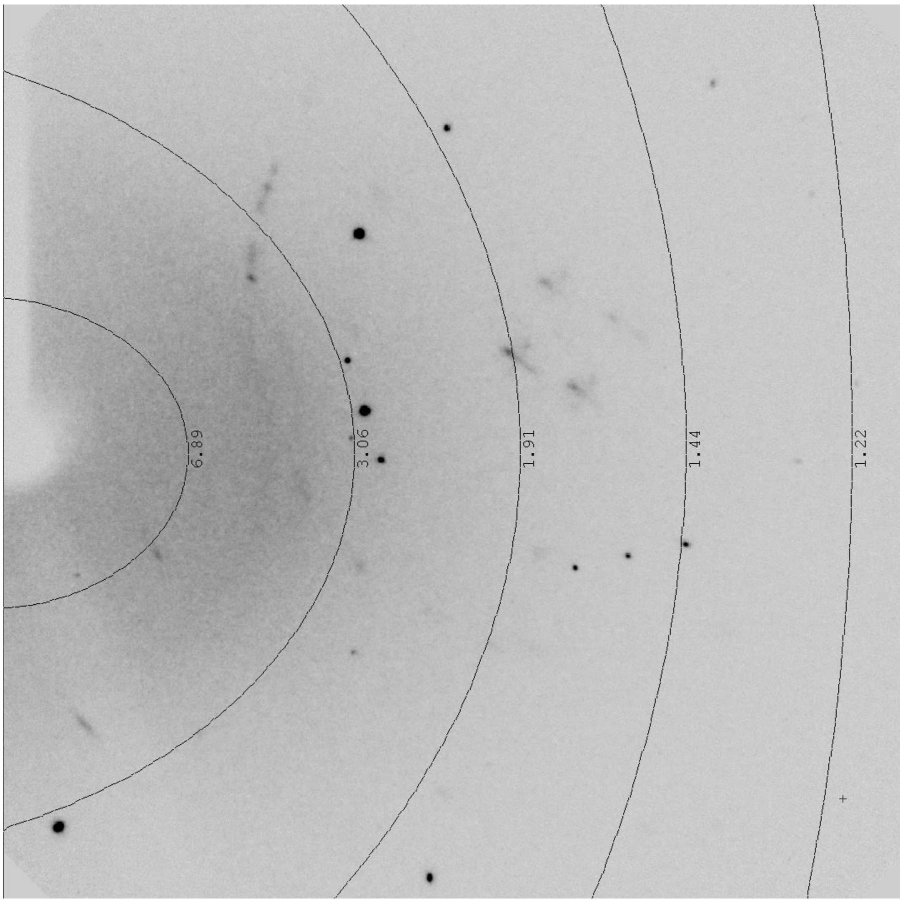

Fig. S 13: Typical frame in the diffraction experiment of **5** (correlated 1° ω-scan with total exposure of 20 sec). The black round dots correspond to the Bragg peaks.

The diffuse spots of the first type lie on the planes perpendicular to ***c****** with the l = integer, near to the points where h and k values are both half-integer. If h or k is integer, the diffuse scattering in the planes is not observed. It is also true for the area near to the systematically absent Bragg reflections with $hk0$, $h+k=2n+1$ (due to the presence of the glide plane $n \perp \mathbf{c}$). The spots of this type have quite a complex shape with two close maximums (Fig. S 10). The distribution of the diffuse scattering intensity is quite narrow but noticeably wider than those for the Bragg peaks (Fig. S 14c-e). The spots of the first types are observed up to high l values (Fig. S 15).

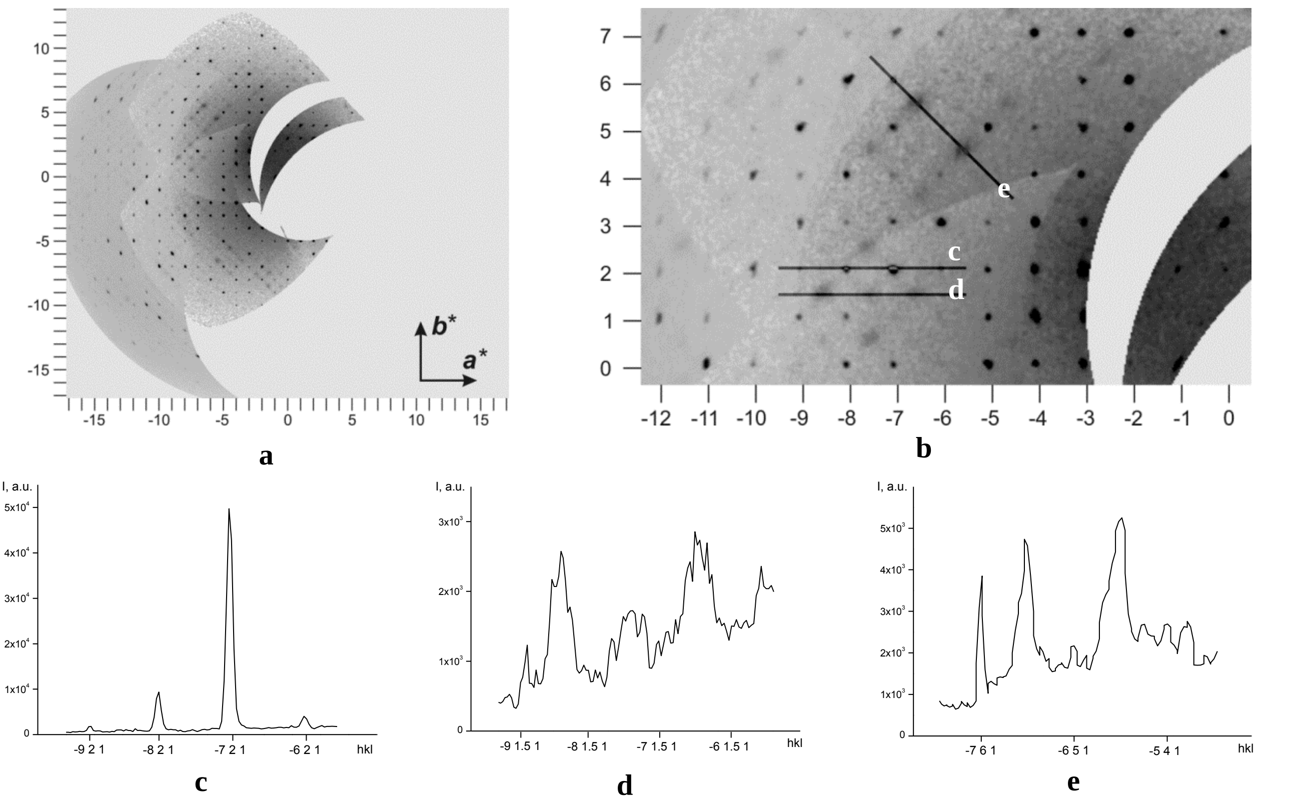

Fig. S 14: The reconstruction of *hk*1 section showing diffuse spots of the first type: general view (a), close view (b) and intensity profile (c, d, e) along the highlited rows at (b).

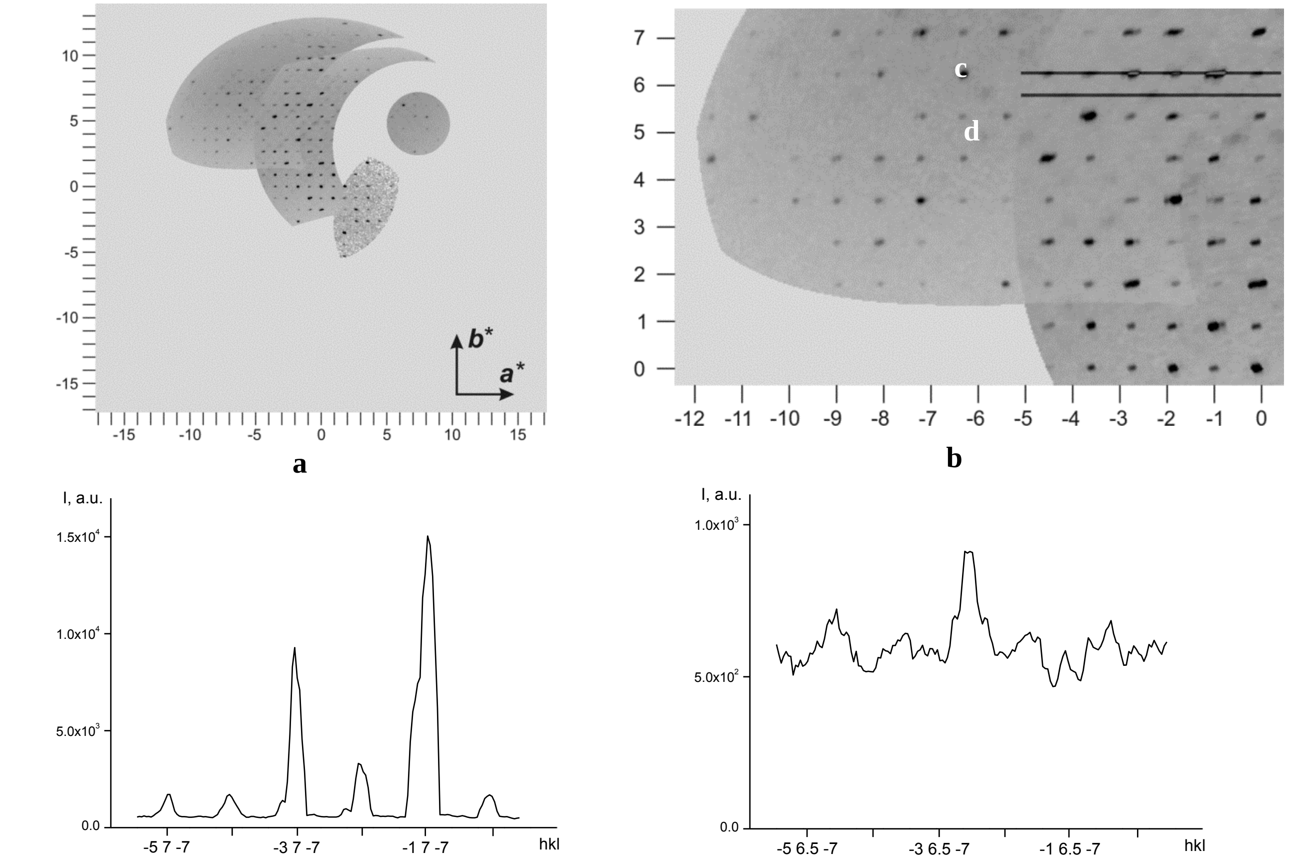

Fig. S 15: The reconstruction of the *hk*-7 section: general view (a), close view (b) and intensity profiles (c, d) along the highlited rows at (b).

The spots of the second type are cross-shaped and lie on the planes perpendicular to ***c****, but on the contrary, with the *half-integer l* and near to the points with *integer h* and *k* values (Fig. S 16). These spots are observed for the whole range -10.5 ≤ *l* ≤ 7.5. The ‘arms’ of the ‘crosses’ are directed along ***a*±b**** diagonals. The reconstruction of the detailed profile for these spots appeared to be impossible due to the artefacts resulting from the wide ω-scans taken during a routine diffraction experiment.

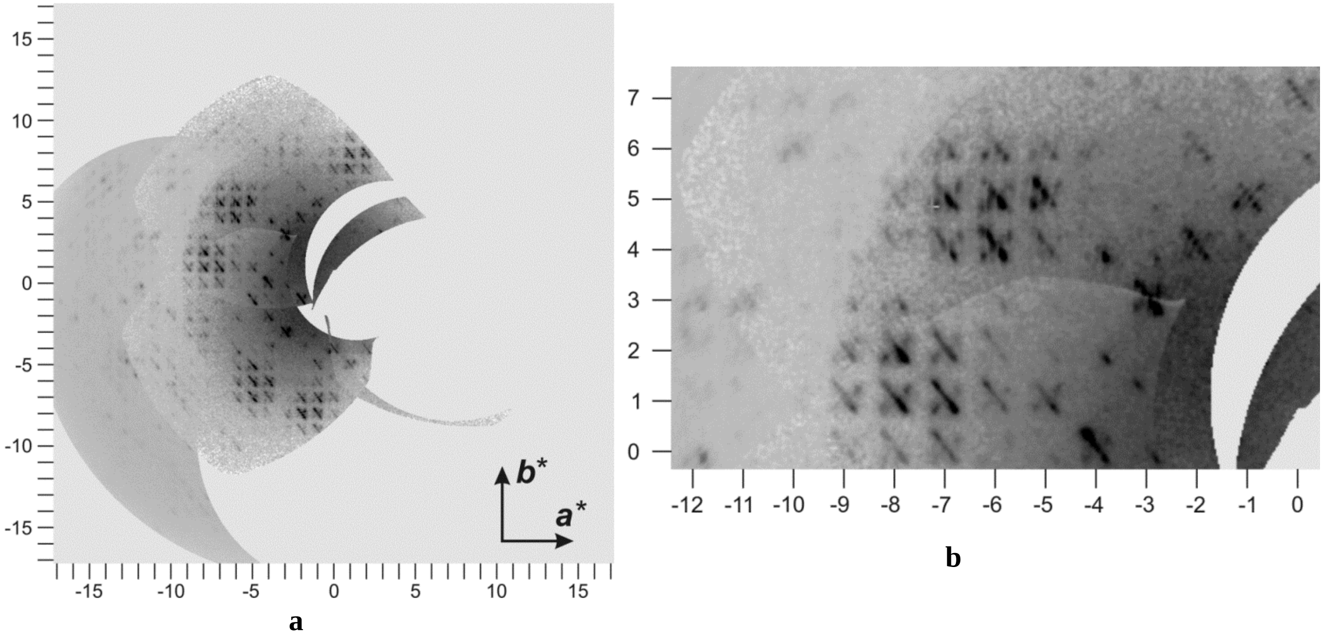

Fig. S 16: The reconstruction of the *hk*0.5 diffraction section: general (a) and close view (b).

The symmetry of the diffuse scattering spots of both types is in a good agreement with the symmetry of the Bragg diffraction (point group 4/*m*).

The diffuse scattering obviously originates from the disorder of the polymeric chains and/or $\{Cu_5I_4(MeCN)_4(P_3C_2Mes_2)\}$ repeating units by 90° rotation around the 4-fold axis parallel to ***c***. At that, each orientation of the $\{Cu_5I_4(MeCN)_4(P_3C_2Mes_2)\}$ repeating unit may require a preferable orientation either of the units of the neighboring chains or within the single chain due to the sterical hindering between the bulky Mes groups. Therefore, there is a correlation between the neighboring orientations despite their equal probability in the averaged crystal structure imposed by the 4-fold axis.

The correct reduction of diffuse intensities for the modelling of the real crystal would require special diffraction techniques (using a strong monochromatic radiation source, noise-free detector *etc*.). The data from a routine structural determination we possess are obviously not enough to make final conclusions. Nevertheless, preliminary consideration shows that we can propose two possible disorder models. One model assumes coexistence of differently oriented chains, but the orientation of all repeating units in every chain is identical. Another more complicated model assumes that the repeating units of the same chain can be rotated around the Cu-P bond in respect to each other. The results of preliminary calculations prove that only the latter model, sophisticated with local relaxation of atomic groups, explains the presence of both types of the diffuse scattering spots. Qualitatively the best result corresponds to the models where the orientation of the closest repeating units alternate both within the polymeric chain and, less strictly, between them.

5. Topological analysis of the 3D polymers

The topological features of the 3D polymers **2**, **8**, **9** were studied with TOPOS 4.0 Professional program.[7] The search in TTO collection implemented in TOPOS gave 8 compounds of the same **hxg** topology deposited in Cambridge Structural Database (corresponding Refcodes are DEGPEL, FUTPUG, KAGCAY, KELJAN, QALHAO, QALHES, VIRPOC and YEMGED). In these complexes of similar structure the {$M_3(\mu_3\text{-}O)$} or {$M_3(\mu_3\text{-}OH)$} units (M^{2+} = Cu^{2+}, Ni^{2+}) join each other by N-heterocyclic bridging ligands, 1,2,4-triazolate,[8] 5-(4-pyridyl)tetrazolate,[9] or pyrazole-4-carboxylate.[10]

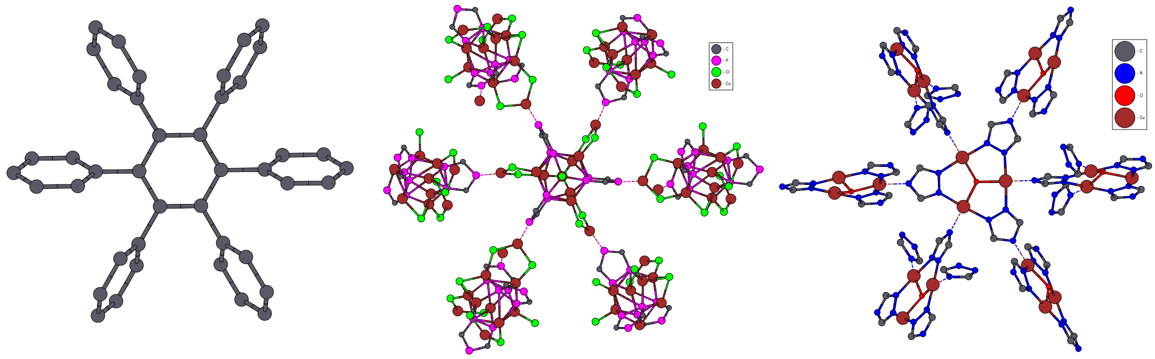

Fig. S 17: Similarity of the structures of (a) polybenzene (calculated)[11] (b) 3D polymer **8** and (c) [$Cu_3(\mu_3\text{-}OH)(\mu_3\text{-}L)(H_2O)_4(OH)_2$], L=1,2,4-triazolate.

6. Pictures of the crystal packing of the polymers 2 - 10

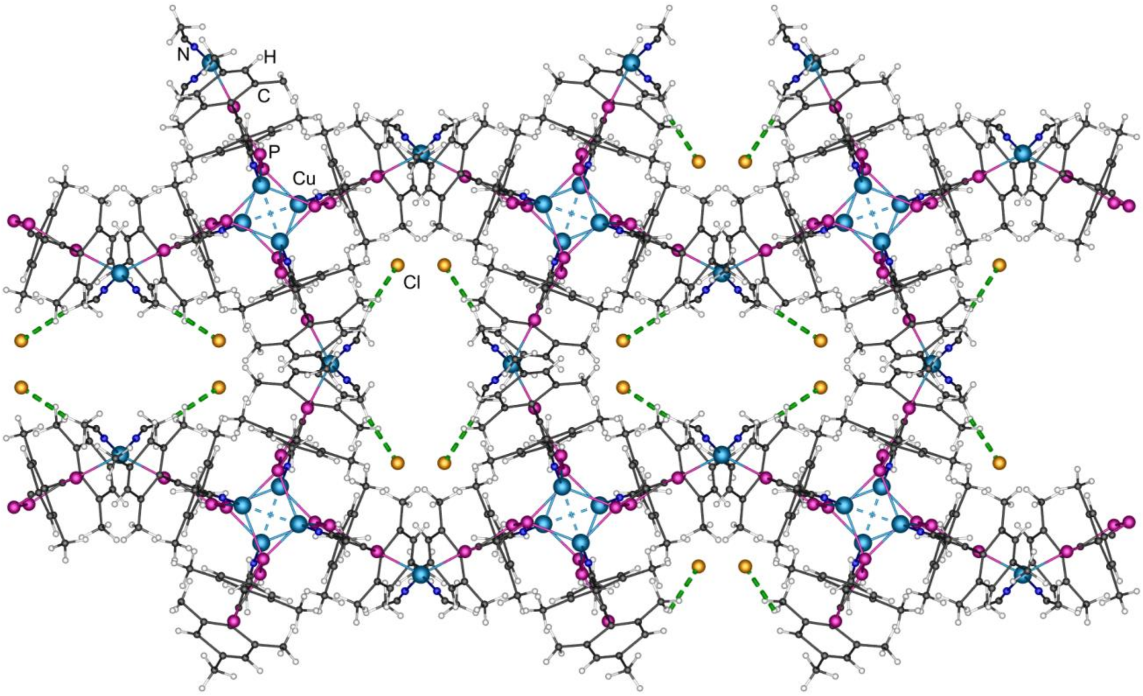

Fig. S18: Section of the polymeric structure of **2**. Short Cl···H distances are illustrated with green dashed lines.

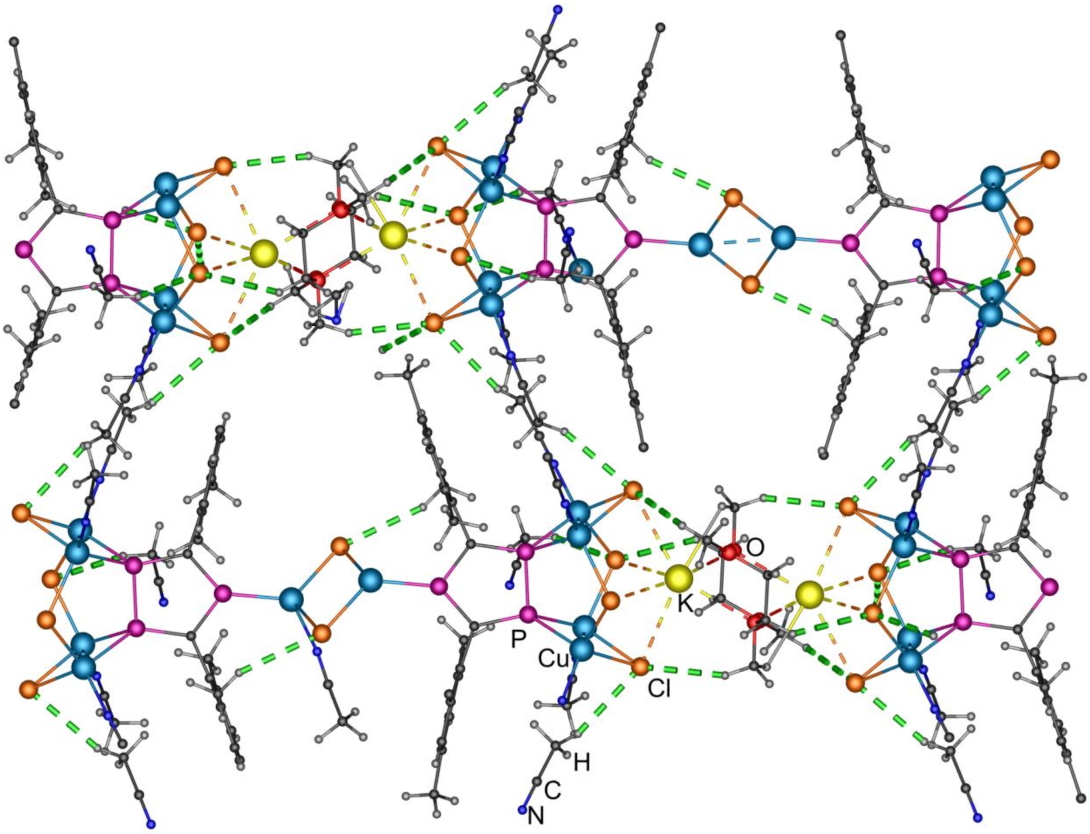

Fig. S19: Section of the polymeric structure of **3**. Short Cl···H distances are illustrated with green dashed lines.

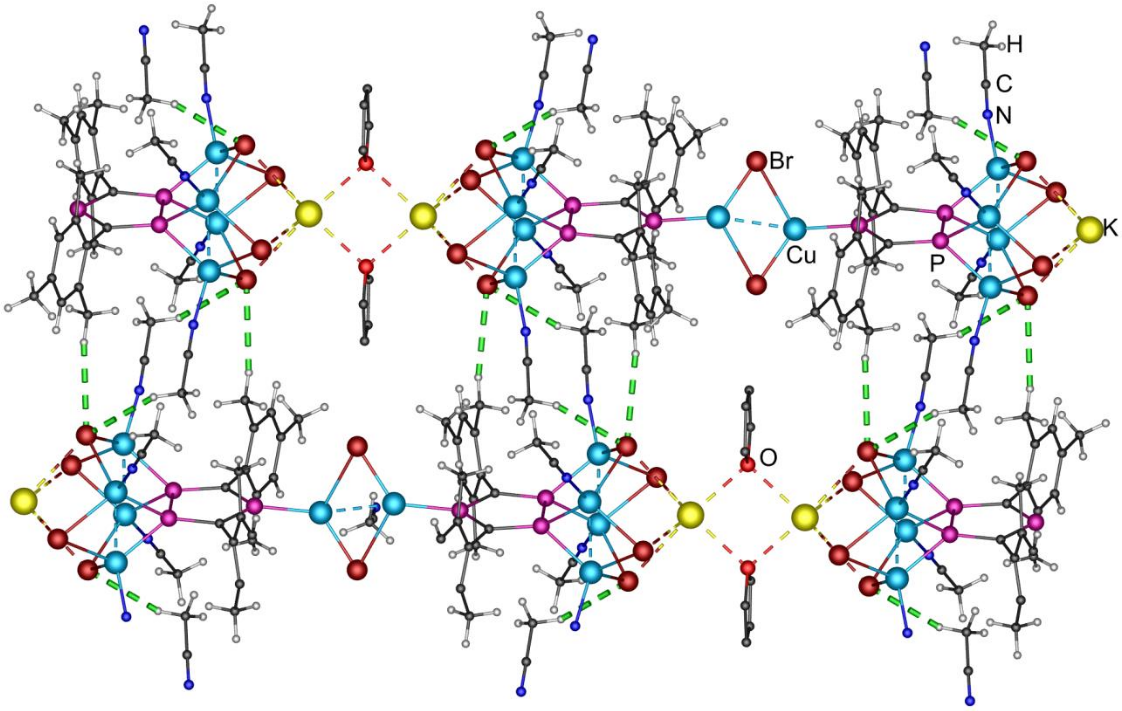

Fig. S20: Section of the polymeric structure of **4**. Short Br···H distances are illustrated with green dashed lines.

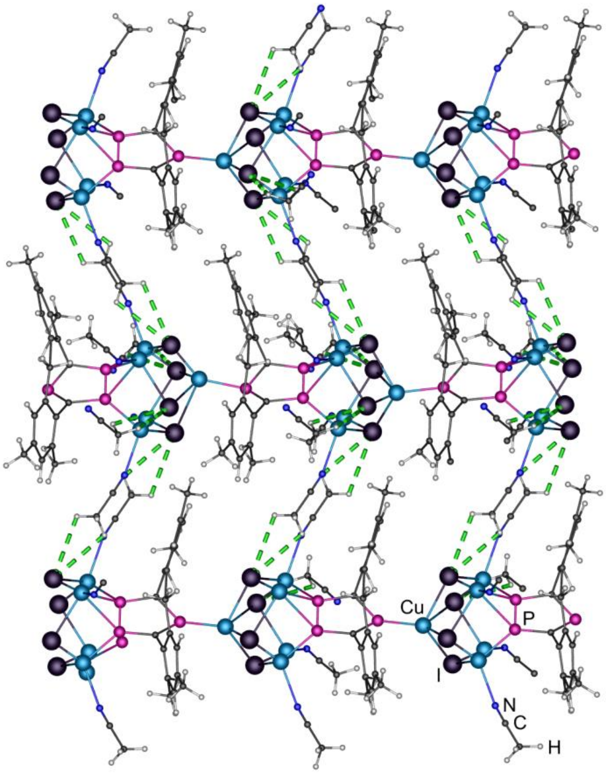

Fig. S21: Section of the polymeric structure of **5**. Short I⋯H distances are illustrated with green dashed lines.

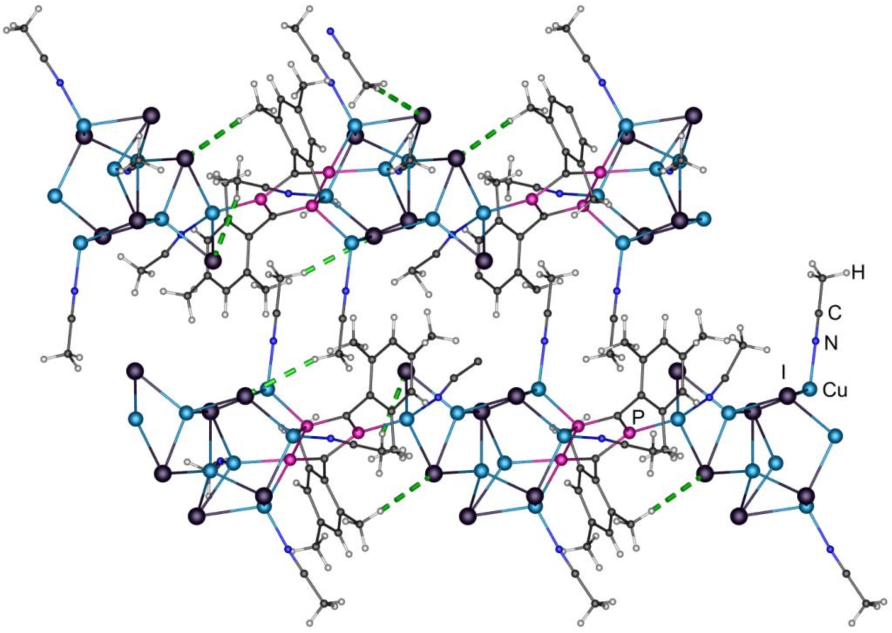

Fig. S22: Section of the polymeric structure of **6**. Short I⋯H distances are illustrated with green dashed lines.

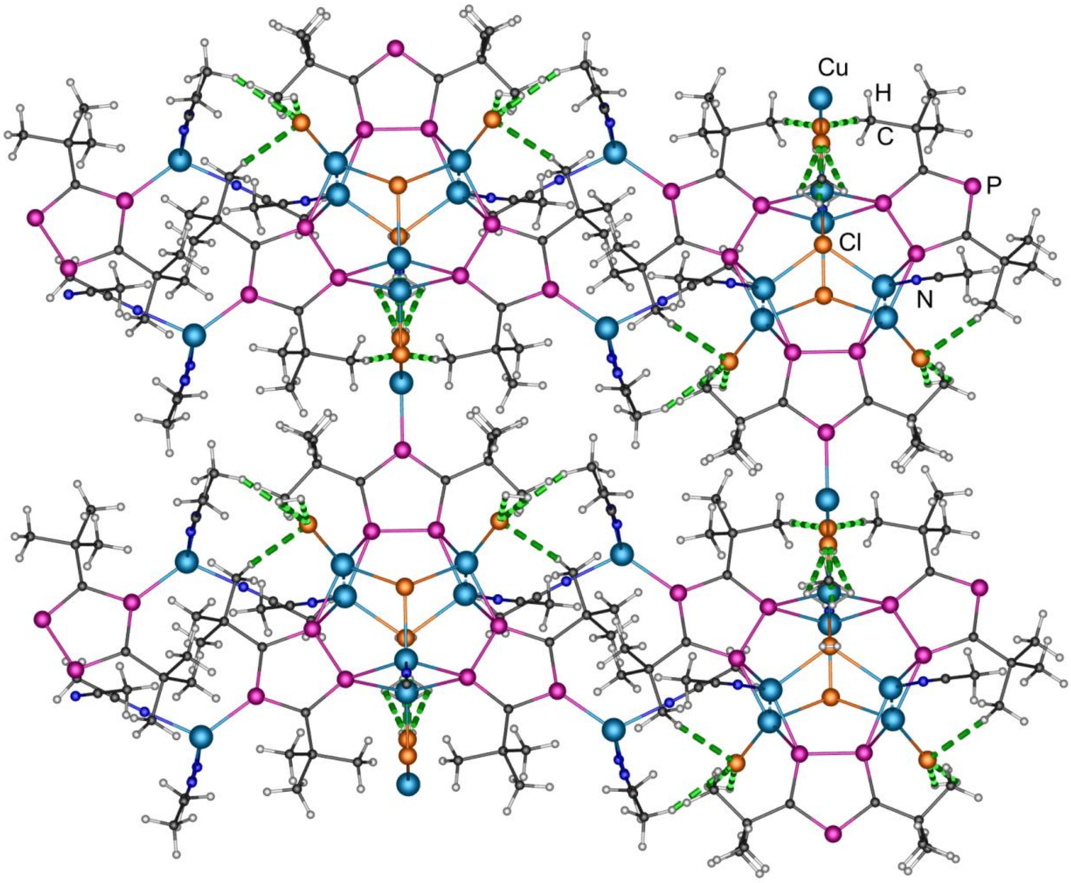

Fig. S23: Section of the polymeric structure of **7**. Short Cl⋯H distances are illustrated with green dashed lines.

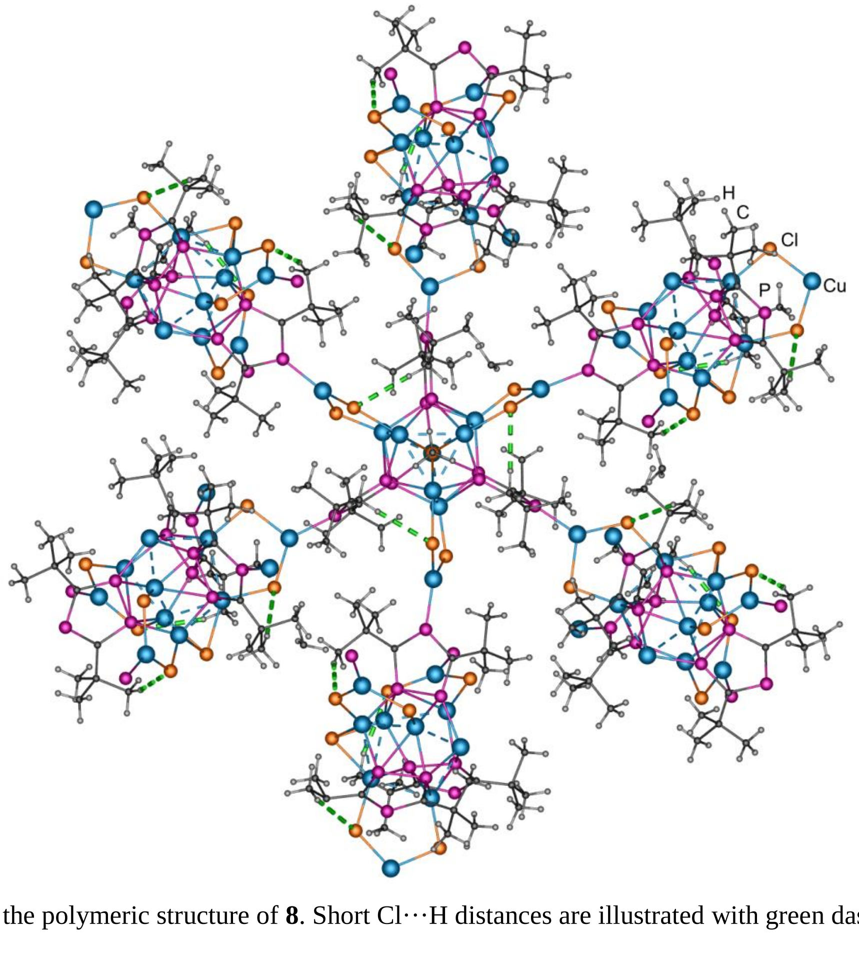

Fig. S24: Section of the polymeric structure of **8**. Short Cl⋯H distances are illustrated with green dashed lines.

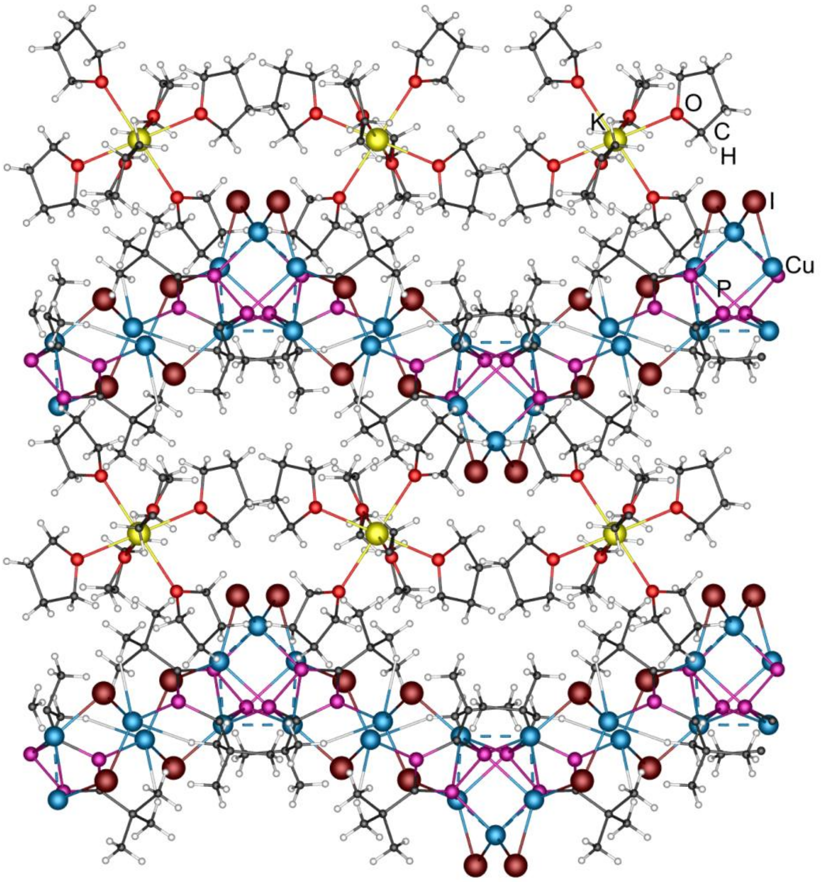

Fig. S25: Section of the polymeric structure of **10** illustrating the alternating cationic and anionic ‘layers’.

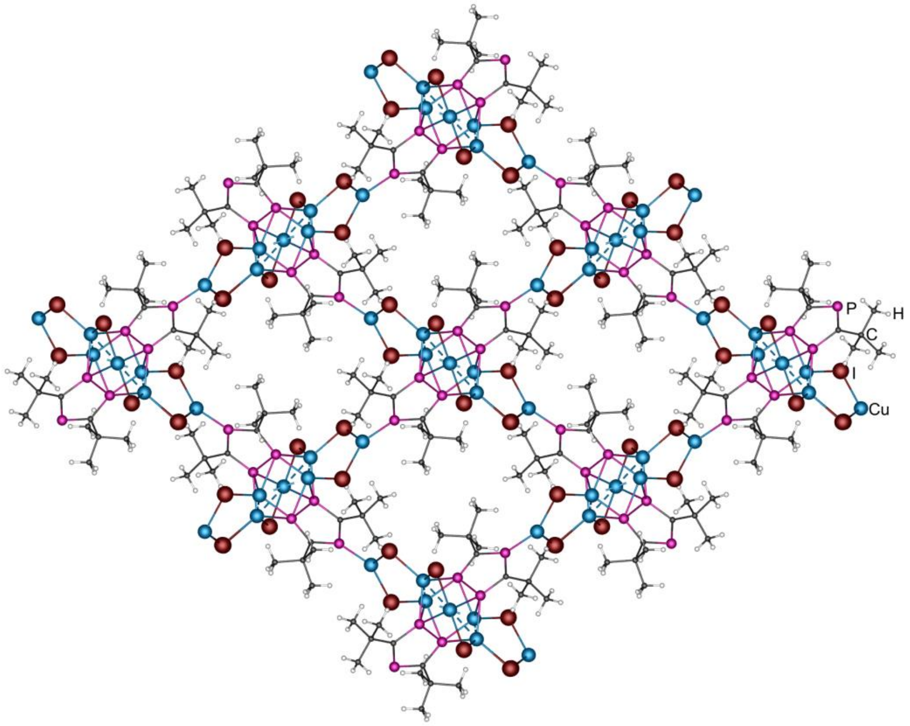

Fig. S26: Section of the polymeric structure of **10** illustrating the mesh-like network.

7. UV-vis Spectroscopy

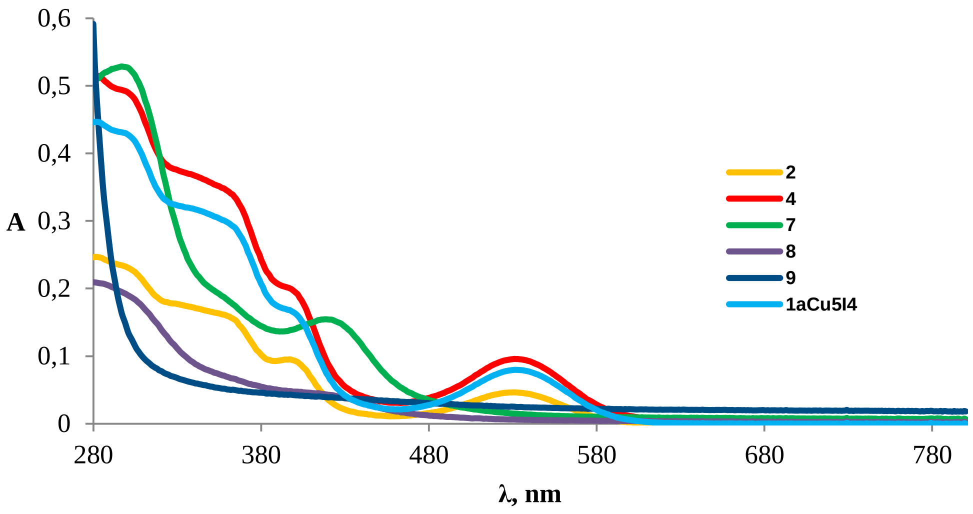

Fig. S27: UV-vis spectra of **2**, **4**, **7-9** and $[(P_3C_2Mes_2)\{Cu(CH_3CN)(\mu_2\text{-}I)\}_4\{Cu(CH_3CN)_3\}]$ in CH_3CN.

Furthermore, solutions of the polymeric compounds **2**, **4**, **7-9** and the monomeric compound $[(P_3C_2Mes_2)\{Cu(CH_3CN)(\mu_2\text{-}I)\}_4\{Cu(CH_3CN)_3\}]$ (marked as **1a**Cu_5I_4) in CH_3CN were analysed by UV-vis spectroscopy. The solubility of the 3D polymer **9** is too poor, even for UV-vis spectroscopy analysis, therefore no absorption could be detected. All other compounds show several partially overlapping absorption bands. The spectra of $[(P_3C_2Mes_2)\{Cu(CH_3CN)(\mu_2\text{-}I)\}_4\{Cu(CH_3CN)_3\}]$, the bromine-containing 1D polymer **4** and the chlorine-containing 3D polymer **2** all show an absorption band at $\lambda = 532$ nm, responsible for the red colour of the respective solution. In contrast, the solutions of the 1D chain **7** and the 3D network **8** are yellow. Therefore no relation between absorption and dimensionality or the present halide of the polymeric scaffolds can be drawn, respectively. However, a relationship between absorption and the R group of the phospholyl group is indicated, since $[(P_3C_2Mes_2)\{Cu(CH_3CN)(\mu_2\text{-}I)\}_4\{Cu(CH_3CN)_3\}]$, **4** and **2** all contain the mesityl substituted building block **1a**, whereas **7** and **8** are built up by the ^{t}Bu derivative **1b**.

References

1 C. Heindl, A. Schindler, M. Bodensteiner, E. V. Peresypkina, A. V. Virovets and M. Scheer, *Phosphorus, Sulfur Silicon and Relat. Elem.* 2015, DOI:10.1080/10426507.2014.979985, *in press.*

2 C. S. J. Callaghan, P. B. Hitchcock, J. F. Nixon, *J. Organomet. Chem.* 1999, **584**, 87.

3 C. Heindl, A. Kuntz, E. V. Peresypkina, A. V. Virovets, M. Zabel, D. Lüdeker, G. Brunklaus and M. Scheer, *Dalton Trans.* 2015, **44**, 6502.

4 G. M. Sheldrick, Acta Cryst. 2015, **C71**, 3.

5 A. Altomare, M. C. Burla, M. Camalli, G. L. Cascarano, C. Giacovazzo, A. Guagliardi, A. G. G. Moliterni, G. Polidori, R. Spagna, *J. Appl. Cryst.* 1999, **32**, 115.

6 Xiu-Ling Li, Xiang-Gao Meng, Suo-Ping Xu, Chin. J. Struct. Chem., 2009, **28**, 1619.

7 V. A. Blatov, A. P. Shevchenko and D. M. Proserpio, *Cryst. Growth Des.* 2014, **14**, 3576.

8 (a) T. Yamada, G. Maruta, S. Takeda, *Chem Commun* 2011, **47**, 653; (b) W. Ouellette, A. V. Prosvirin, J. Valeich, K. R. Dunbar, J. Zubieta, *Inorg. Chem.* 2007, **46**, 9067; (c) W. Ouellette, M. H. Yu, C. J. O'Connor, D. Hagrman, J. Zubieta, *Angew. Chem., Int. Ed.* 2006, **45**, 3497; (d) Q.-G. Zhai, C.-Z. Lu, S.-M. Chen, X.-J. Xu, W.-B. Yang, *Cryst. Growth Des.* 2006, **6**, 1393; (e) B. Ding, L. Yi, P. Cheng, D.-Z. Liao, S.-P. Yan, *Inorg. Chem.* 2006, **45**, 5799.

9 W. Ouellette, H. Liu, C. J. O'Connor, J. Zubieta, *Inorg. Chem.* 2009, **48**, 4655.

10 E. Quartapelle Procopio, F. Linares, C. Montoro, V. Colombo, A. Maspero, E. Barea, J. A. R. Navarro, *Angew. Chem., Int. Ed.* 2010, **49**, 7308.

11 M. O'Keeffe, G. Adams, O. Sankey, *Phys. Rev. Lett.* 1992, **68**, 2325.
